# Thermodynamic basis for the demarcation of Arctic and alpine treelines

**DOI:** 10.1038/s41598-022-16462-2

**Published:** 2022-07-22

**Authors:** Meredith Richardson Martin, Praveen Kumar, Oliver Sonnentag, Philip Marsh

**Affiliations:** 1 Department of Civil and Environmental Engineering, University of Illinois at Urbana-Champaign, Urbana, 61801 USA; 2 Department of Atmospheric Sciences, University of Illinois at Urbana-Champaign, Urbana, 61801 USA; 3 Centre d’études nordiques, Université de Montréal, Montréal, QC H2V 2B8 Canada; 4 Cold Regions Research Centre, Wilfrid Laurier University, Waterloo, ON N2L 3C5 Canada

**Keywords:** Ecological modelling, Hydrology

## Abstract

At the edge of alpine and Arctic ecosystems all over the world, a transition zone exists beyond which it is either infeasible or unfavorable for trees to exist, colloquially identified as the treeline. We explore the possibility of a thermodynamic basis behind this demarcation in vegetation by considering ecosystems as open systems driven by thermodynamic advantage—defined by vegetation’s ability to dissipate heat from the earth’s surface to the air above the canopy. To deduce whether forests would be more thermodynamically advantageous than existing ecosystems beyond treelines, we construct and examine counterfactual scenarios in which trees exist beyond a treeline instead of the existing alpine meadow or Arctic tundra. Meteorological data from the Italian Alps, United States Rocky Mountains, and Western Canadian Taiga-Tundra are used as forcing for model computation of ecosystem work and temperature gradients at sites on both sides of each treeline with and without trees. Model results indicate that the alpine sites do not support trees beyond the treeline, as their presence would result in excessive CO$_2$ loss and extended periods of snowpack due to temperature inversions (i.e., positive temperature gradient from the earth surface to the atmosphere). Further, both Arctic and alpine sites exhibit negative work resulting in positive feedback between vegetation heat dissipation and temperature gradient, thereby extending the duration of temperature inversions. These conditions demonstrate thermodynamic infeasibility associated with the counterfactual scenario of trees existing beyond a treeline. Thus, we conclude that, in addition to resource constraints, a treeline is an outcome of an ecosystem’s ability to self-organize towards the most advantageous vegetation structure facilitated by thermodynamic feasibility.

## Introduction

Explaining the heterogeneous organization of vegetation across landscapes has proved both a puzzling and an inspiring concept as patterns have formed naturally across the world. One such pattern is the existence of treelines, i.e., the demarcation zone between forestland and vegetation without trees^1,2^. There is a large body of work with developed and competing theories for understanding the specific limits and drivers for the non-existence of trees beyond a treeline. Yet after decades of study, there is still debate among ecologists and biologists over the mechanisms that limit the presence of trees beyond treelines. Current explanations are rooted in, but not limited to, consideration of factors such as excessive light and wind, limited CO$_2$, and low temperatures^1,3–7^.

With this in mind, we ask: *Is there another perspective that could provide insights complementary to and beyond what has been developed through the prevailing mechanistic approach?* While the existing explanations are based on ideas of structural stability (e.g., high winds above the treeline) and limited resources pertaining to water, energy, and nutrients^1,3–7^, we instead examine the question of what determines the existence of a treeline from the perspective of thermodynamic feasibility. Our premise is that the existence or non-existence of certain vegetation first and foremost has to be ascertained through thermodynamic feasibility or infeasibility, respectively. Therefore, we approach the question of the existence of treelines by asking: *If certain vegetation does not exist at a given location, is there a role that the thermodynamic perspective can play in telling us that its existence is infeasible?* By approaching the topic from the thermodynamic perspective, we seek to provide important complementary insight to the broad base of scientific understanding ecologists and biologists have developed to explain the existence of treelines. Further, this work lays out additional context for the discussion around the advance of treelines (e.g., why some treelines advance and others do not).

For example, several theories assert that the stature of vegetation is limited by CO$_2$ balance and photosynthetic requirements under harsh winter conditions^1,6^. Other hypotheses argue that plant life is instead limited by the atmospheric temperature and the local environments that the plants experience^7,8^. *Is there a perspective that could unify both of these findings?* Through this work, we demonstrate how thermodynamic infeasibility inferred from model simulations pertaining to counterfactual scenarios manifests through both of these physiological limits. This means that either of these limits, individually or together, could lead to the nonexistence of trees—which limiting factor is expressed first varies by location. Thus, the commonality among locations that have different limiting mechanisms can be found in the unifying concept of thermodynamic infeasibility. While CO$_2$ limitation may prevail in one location and temperature-related constraints may be limiting in another, both lead to thermodynamic infeasibility, meaning that the thermal environment results in a mechanistic infeasibility, such as net CO$_2$ loss. In the examples presented in this paper, thermodynamic infeasibility manifests through negative work associated with constraints arising from temperature gradients and net CO$_2$ loss, demonstrating that both limitations can be encapsulated using the thermodynamic perspective.

### Ecosystem thermodynamics

It is now generally accepted that observed patterns of vegetation composition and its organization are a result of self-organization, or the spontaneous emergence of pattern without external predetermination^9,10^. By framing ecosystems as open thermodynamic systems, we explore further the concept of thermodynamic feasibility and its role in the self-organization of vegetation structure. Vegetation structure consists of composition (i.e., the number and type of functional groups^11^) and organizational patterns on the landscape^12^. We focus on composition rather than the spatial pattern of vegetation organization. We utilize a one-dimensional ecohydrological model that incorporates representative functional groups with no lateral transport of energy or matter under the assumption that the vegetation composition and pattern remain spatially uniform at a given site. Thus, we are able to compare the vertical thermodynamic regimes of proximal ecosystems with varying vegetation composition. We present the case that observed organization reflected in the demarcation of differing vegetation structures on either side of a treeline is established in tandem with vertical thermodynamic gradients at a given location, driven by the incoming solar energy into an ecosystem. In other words, *we hypothesize that beyond a treeline, the existence of trees is prevented by conditions of thermodynamic infeasibility.*

The application of thermodynamic theory to ecology has been studied for the better part of the last century through the introduction of theoretical thermodynamic properties, such as entropy and exergy, into environmental systems. This work asserts that open thermodynamic systems will evolve based on the strength of applied concentration gradients on the system and will undergo irreversible processes to dissipate energy and destroy these gradients through all means available^13,14^. In the context of ecosystems, fluxes of mass or energy from the external environment (i.e., above the canopy) result in concentration gradients within the system itself. State variables will transition along these gradients according to the second law of thermodynamics. When the magnitude of incoming energy and consequent spatial imbalance of energy becomes great enough, dissipative structures spontaneously emerge, or self-organize, and establish temperature gradients consistent with the dissipative need of the ecosystem^13,15^. In this paper we conceptualize the work performed by an ecosystem as its ability to dissipate these applied concentration gradients. Consequently, work is highly dependent upon the existence and composition of self-organized vegetation.

In classical thermodynamics, work is performed due to a transfer, or physical movement, of heat^15^. In the context of ecosystems, work performed by an ecosystem is represented by the exchange of heat with the external environment outside the ecosystem control volume^12^. Work performed by an ecosystem is, therefore, estimated as the vertical transport of heat in the form of latent and sensible heat, driven by the vertical gradient in temperature within the control volume structured by both the incoming downward shortwave and longwave radiation and the vegetation structure. The bottom boundary of the ecosystem control volumes studied are significantly deep such that heat exchanges due to water infiltration at this interface are insignificant in magnitude relative to latent and sensible heat flux out of the top of the control volume above the canopy. Further, we ignore the substantially slower thermodynamic processes associated with geochemistry in the soil.

The vertical temperature gradient creates a directionality of dissipation of incident radiation as heat leaves out of the ecosystem from higher surface temperatures to lower air temperatures. Throughout this paper, we measure work through the net sum of heat leaving the ecosystem as latent and sensible heat—which can either be positive or negative depending on the direction of the resultant temperature gradient (see “Thermodynamic Calculations” in the “Methods” section). This temperature gradient (Eq. 1) emerges as a result of self-organization through feedback between the incoming shortwave and longwave radiation, local environmental conditions, and the heat dissipation and work performed by the vegetation. The presence of ground cover, such as snow, is impacted by aboveground vegetation structure, which provides a physical buffer between the atmosphere and the ground, further influencing the thermal environment and temperature gradient.

Although significant research has been conducted by studying plant response to snowpack^7,16,17^, including the physiological requirements for life under prolonged snowpack and alpine climatic conditions, the thermodynamic perspective provides further insight. In addition to the physiological/mechanistic response of plants to snowpack and other environmental conditions, the thermal regime of a column of land experiencing snowpack is fundamentally different when an ecosystem does or does not have plants with stature taller than the height of snowpack (e.g., trees). Presence of trees results in shading from solar radiation and a physical buffer between the earth/snow surface and the atmosphere. Thus, the thermal profile of an ecosystem reveals valuable information about ecosystem behavior, and there is a need to explore the thermodynamic relationship between solar radiation and vegetation composition under varying environmental conditions. Thus, through this paper we define the circumstances under which multiple functional groups that include trees are no longer feasible for the available solar radiation leading to demarcated zones identifiable as treelines.

Work by Körner argues that the “climate [that] plants experience” is different than the ambient temperature^7^. By modeling the layers within the canopy of plants with differing stand heights and leaf distributions, we are able to characterize the thermal regime and the “climate [that] plants experience” throughout the course of a given year. This characterization helps us understand the fundamental changes in behavior under varying environmental conditions with and without trees.

**Figure 1:**
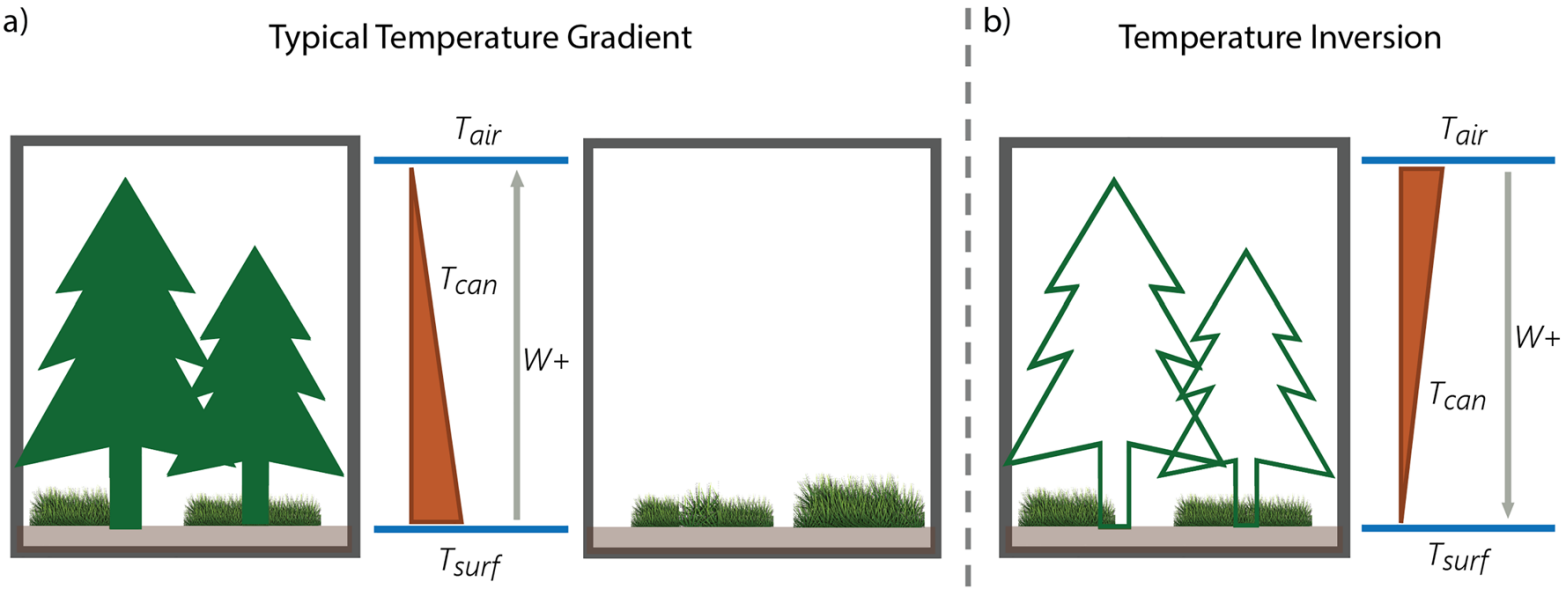
Conceptual diagram of temperature gradients. The *W+* arrow indicates the positive direction of work performed through heat transport. Although in different directions, in both cases (**a**) and (**b**), the work performed is positive because heat moves from high to low temperatures. (**a**) Typical summertime temperature gradients from the earth surface to the air above the canopy are negative for the two real scenarios: subalpine/sub-Arctic forest (*left*) and alpine tundra/Arctic meadow (*right*). (**b**) A conceptual temperature inversion, or positive temperature gradient, which arise when alpine/Arctic forest are simulated as counterfactuals.

An ecosystem’s ability to perform work manifests into four distinct cases depending on the sign of the resultant temperature gradient and the net loss or gain of heat driven by the thermal environment derived from present ground cover, such as vegetation or snow: (1) First and most common during the day when photosynthesis is occurring, the temperature of the earth surface, which receives the solar radiation, is typically warmer than the air above the canopy, and heat leaves the ecosystem upward along the negative temperature gradient, corresponding to a positive work (Fig. 1a). (2) Even when the temperature of the earth surface is warmer than the air above the canopy, there can be situations when there is a net heat gain within the ecosystem, meaning that heat moves into the ecosystem against the direction of the temperature gradient. This case is rare and counterproductive to heat dissipation, corresponding to negative work. (3) Common during the night, temperature inversion emerges. In this case, the temperature gradient from the earth surface to the atmosphere can become positive, meaning that the temperature of the air above the canopy is greater than the temperature of the earth surface. As heat enters the ecosystem to warm the surface, positive work is performed since the heat is still moving along a negative temperature gradient into the ecosystem (Fig. 1b). (4) During snowmelt conditions during the day, particularly for Arctic and alpine ecosystems, temperature inversions also emerge^18,19^. When this occurs and the ecosystem experiences a net heat loss through latent and sensible heat from the canopy, the heat leaving the ecosystem travels opposite of the direction dictated by the temperature gradient. Thus, in this case, ecosystems perform negative work. Our findings demonstrate how extended periods of time in this last case of work lead to thermodynamic infeasibility for the alpine/Arctic ecosystem counterfactual vegetation scenarios; i.e., ecosystems with vegetation properties from below the treelines cannot be sustained under the environmental conditions above the treelines, and, hence, they do not occur in nature.

A recent study concluded that at sites where multiple functional groups exist (e.g., forests), the vegetation structure in which all groups co-exist and interact is thermodynamically more advantageous and, thus, more likely to occur than any one of the individual functional groups that the forest comprises^12^. Thermodynamic advantage is defined by the production of larger fluxes of entropy, more work performed, and higher work efficiency – a quantity that captures how much of the incoming energy is converted into forms useful for actively dissipating heat. It is possible to envision that under certain environmental conditions, the thermodynamic advantage offered by the existence of multiple functional groups is not tenable, indicating a thermodynamic infeasibility. Thermodynamic infeasibility occurs when a particular vegetation structure is not supported by the thermal environment at a given location. The demarcation exhibited by treelines presents an ideal case to explore this scenario, in that there is a distinct transition from multiple functional groups below the treeline to a single functional group above.

### Research question

In this paper, we examine vegetation above and below Arctic and alpine treelines to determine whether the absence of trees in ecosystems above treelines are a result of thermodynamic infeasibility. Simply speaking, we seek to answer the following research question: *Is the non-existence of trees beyond the transition zone demarcated as a treeline a reflection of thermodynamic infeasibility associated with the presence of trees, and if so, how is this infeasibility exhibited?*

**Figure 2:**
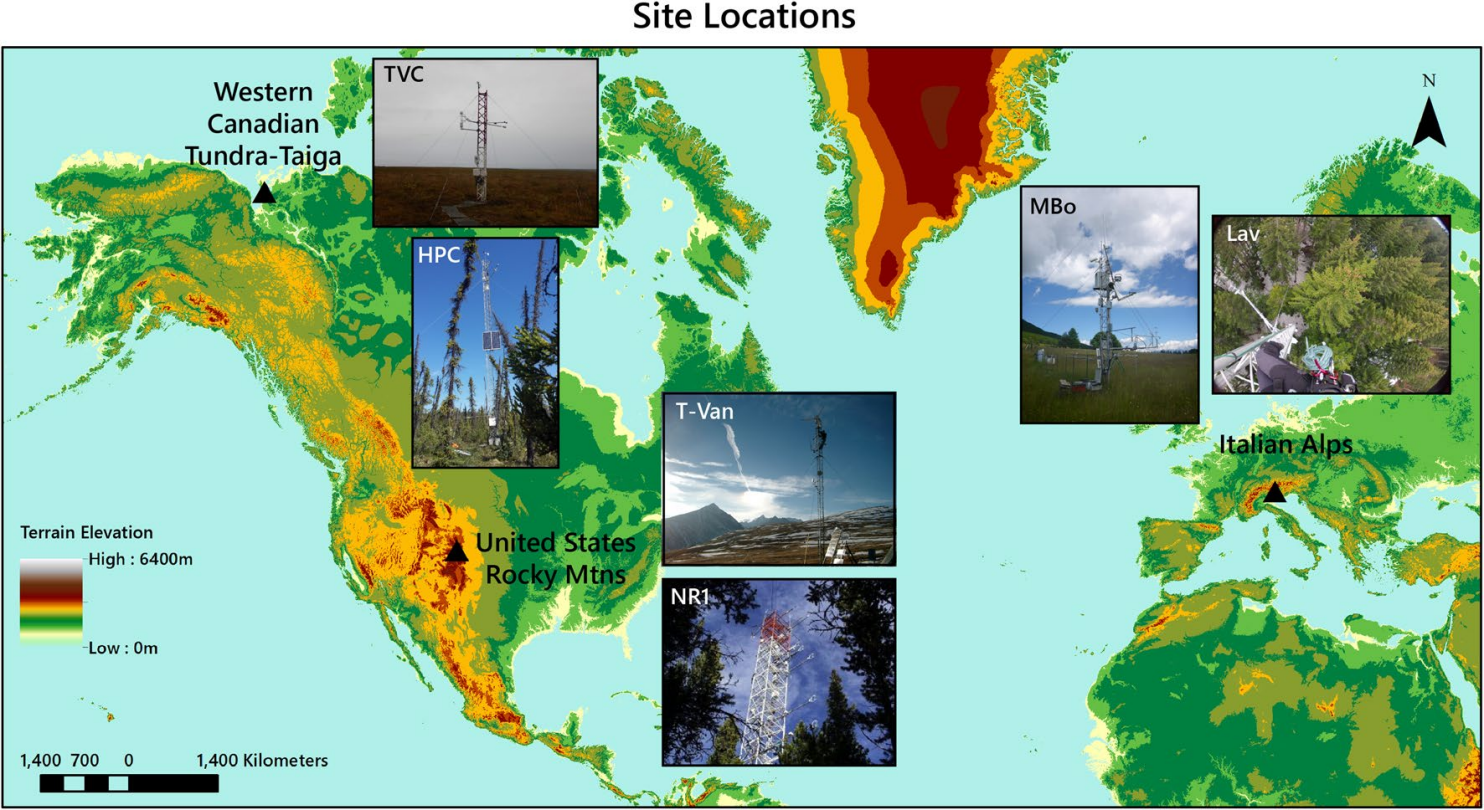
Site locations for the three pairs of sites (Table 1) above and below Arctic and alpine treelines. The background topographic map is generated from NASA space-based elevation data (https://visibleearth.nasa.gov/images/73934/topography#). Fluxtower site images were obtained from the following: TVC & HPC—Oliver Sonnentag (https://atmosbios.com/); T-Van—photo of the Saddle site about 350m along the ridge from T-Van taken by Andy Watt (https://archive.eol.ucar.edu/homes/stephens/RACCOON/NWRsite.html); NR1—Sean Burns, 10/07/2014, (https://ameriflux.lbl.gov/sites/siteinfo/US-NR1); MBo & Lav—Roberto Zampedri, Fondazione Edmund Mach.

To address this question, we use an extensively validated multi-layer 1-D physics-based ecohydrological model, MLCan^12,20–26^, consisting of 20 above-ground layers, 1 ground surface layer, and 12 below-ground layers (see *Supplementary Material*). This model is chosen because of its ability to capture interactions among functional groups, such as the impact of shading on understory vegetation and the resulting thermal environment within the canopy^23^. To balance model performance and accuracy, standing plant species are aggregated into functional groups (i.e., evergreen needleleaf trees, shrubs, grasses; see Table 1) based on literature^27–30^. The model output is used to compare the thermodynamic work performed at paired sites above and below the respective treelines for three different locations: the Italian Alps (IT), the United States Rocky Mountains (US), and the Western Canadian Taiga-Tundra (CA) (Fig. 2; see Site Descriptions). For each site pair, four scenarios are performed (Table 1): (1) The subalpine/sub-Arctic forest ecosystems are modeled as they exist with multiple functional groups (Fig. 1a, *left*). (2) The alpine/Arctic ecosystems are modeled as they exist with one functional group (i.e., shrubs or grasses; Fig. 1a, *right*). (3) We construct counterfactual scenarios above the treeline in which the vegetation of the subalpine or sub-Arctic forest is simulated with the environmental conditions and parameters of the alpine meadow or Arctic tundra (i.e., adding hypothetical trees where none exist; Fig. 1b). (4) As a control, a final counterfactual scenario is constructed below the treeline in which we model the understory of the subalpine/sub-Arctic forest individually (i.e., removing trees from the existing ecosystem).

**Table 1:** Simulation scenarios with observed and hypothetical vegetation.

Abbrev.	UN FG	OT FG	Counterfactual?	Site ID
**Italian Alps**				
IT-Alp	grasses	–	no	MBo
IT-Tr	suppr DT	ENT	yes	MBo
IT-For	suppr DT	ENT	no	Lav
IT-Un	suppr DT	–	yes	Lav
**United States Rocky Mountains**				
US-Alp	sedge	–	no	T-Van
US-Tr	shrubs	ENT	yes	T-Van
US-For	shrubs	ENT	no	NR1
US-Un	shrubs	–	yes	NR1
**Western Canadian Taiga-Tundra**				
CA-Arc	shrubs	–	no	TVC
CA-Tr	shrubs	ENT	yes	TVC
CA-For	shrubs	ENT	no	HPC
CA-Un	shrubs	–	yes	HPC

The simulation abbreviations are defined as ‘location’-‘scenario’, where the locations are the Italian Alps (IT), United States Rocky Mountains (US), and the Western Canadian Taiga-Tundra (CA); and the scenarios are defined as follows: Alp—alpine meadow or fellfield, Arc—Arctic tundra, Tr—alpine/Arctic site with simulated trees, For—subalpine/sub-Arctic forest, Un—subalpine/sub-Arctic forest understory simulated without overstory trees. Throughout this paper, ‘X-’ is used to represent all locations (e.g., X-Un encompasses IT-Un, US-Un, and CA-Un). The understory (UN) and overstory (OT) functional groups (FG) for each modeled scenario are identified based on the Site IDs described in the *Site Descriptions* section of the “Methods”. ‘suppr DT’ refers to suppressed deciduous trees, and ‘ENT’ refers to evergreen needleleaf trees. The ‘Counterfactual’ column indicates whether synthetic vegetation was simulated.

The simulation of these four scenarios facilitates comparison of the existing vegetation structure of each site with the corresponding counterfactual scenarios. By varying the model inputs of vegetation present at each site while holding the environmental conditions and site-specific parameters consistent, we are able to directly compare thermodynamic outcomes as a result of varying vegetation structure and determine whether the counterfactual scenario with the simulated forest is thermodynamically feasible. Model performance was judged based on comparison to observed heat fluxes, such as latent and sensible heat (see *Supplementary Material*, Figs. S1–S3). As detailed below, the analysis supports the conclusion that thermodynamic feasibility is an important and complementary condition to the usual considerations of resource availability, such as water and nutrients, which determines the organizing form and function of ecosystems.

## Results

Model simulations from the three site pairs highlight two primary conditions of thermodynamic infeasibility that helps explain the non-existence of trees beyond a treeline. The first thermodynamic infeasibility is associated with alpine/Arctic temperature inversions (i.e., positive temperature gradients from the earth surface to the atmosphere), which result in prolonged periods of negative work (i.e., heat leaving the ecosystem against the temperature gradient) for the counterfactual tree scenarios for all three locations. The second manifests in the counterfactual alpine tree scenarios, (IT-Tr and US-Tr) as temperature-driven feedbacks lead to a decrease in net CO$_2$ gain or unsustainable net CO$_2$ loss from the vegetation’s leaves for the two alpine sites.

In this section, we elucidate the relationship of work with temperature gradient; highlight the two conditions of thermodynamic infeasibility through site examples; and illustrate how these conditions of infeasibility translate into thermodynamic limits that explain the self-organization of differing vegetation structure on either side of a treeline.

### Negative work

**Figure 3:**
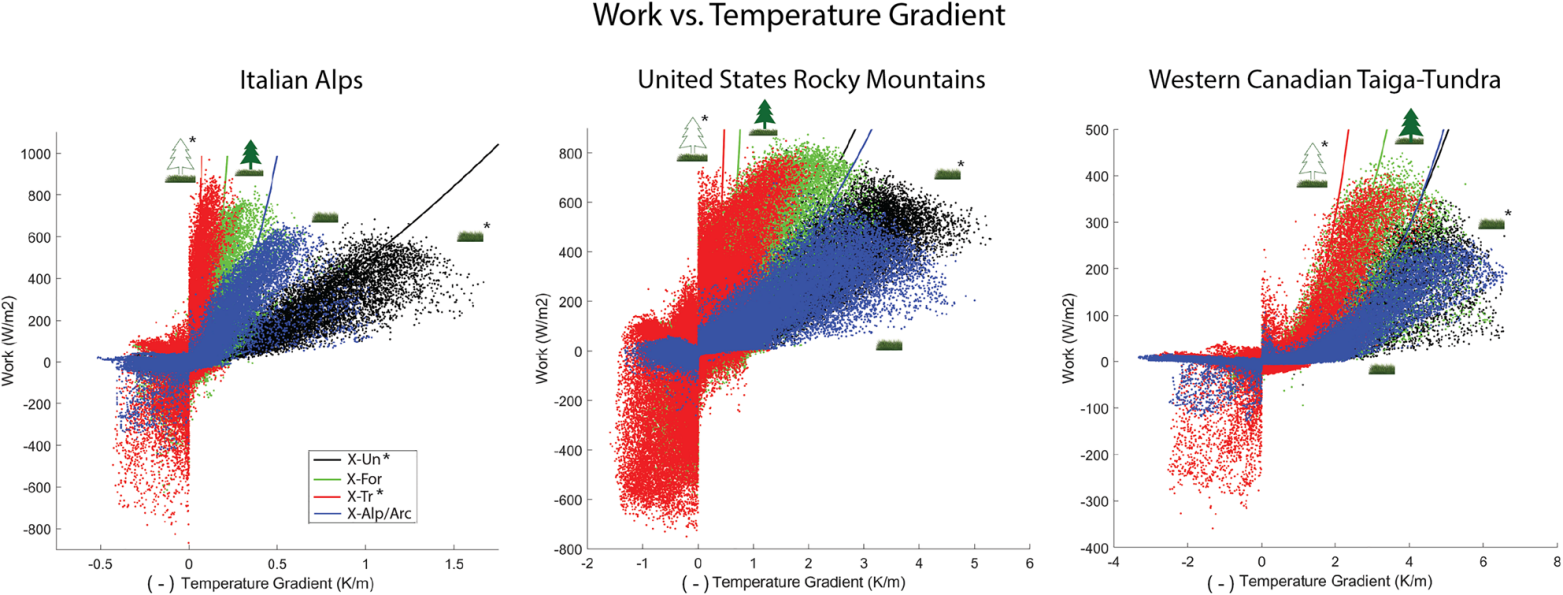
The work versus resultant temperature gradient for the three site pairs summarized in Table 1. The negative of the resultant temperature gradient is plotted on the x-axis. Thus, the positive x-axis refers to a negative temperature gradient such that larger values indicate stronger declines in temperature from the earth surface to the atmosphere. The negative x-axis indicates positive temperature gradients, or temperature inversions. Data points represent half-hourly simulation timesteps over the entire study period (Italy, 2 years; United States, 6 years; Canada, 3 years). The starred vegetation scenarios (X-Un* & X-Tr*) indicate counterfactual scenarios. For every region, on the positive x-axis the simulated trees (*X-Tr*) scenario above the treeline performs the most work for the resultant temperature gradient than the other scenarios when the temperature of the earth surface is warmer than that of the air above the canopy. However, when the resultant temperature gradient is positive (negative x-axis), a temperature inversion occurs (Fig. 1), resulting in the vegetation performing negative work – transporting heat in the opposite direction of the temperature gradient (*X-Tr* & *X-Alp/Arc*).

By visualizing the work performed through dissipation of heat for a resultant temperature gradient, we are able to assess the thermodynamic feasibility of the various scenarios for each ecosystem. Figure 3 displays the work performed versus the resultant temperature gradient at each half-hourly model timestep for all sites and scenarios. First considering the scenarios below the treeline, the subalpine/sub-Arctic forest (*green*) is more thermodynamically advantageous than the simulated understory alone (*black*) for each location. This advantage is demonstrated by greater work performed by the *X-For* scenarios (i.e., IT-For, US-For, and CA-For; see Table 1) per unit increases in resultant temperature gradient, indicating that the existing forest vegetation self-organizes its thermal environment such that it more rapidly dissipates heat, moving it away from the ecosystem into the atmosphere. This result is consistent with conclusions from previous work^12^ that the co-existence of multiple functional groups in natural forested ecosystems is more thermodynamically advantageous than each of the individual groups comprising the forest vegetation structure.

For the subalpine/sub-Arctic sites, resultant temperature gradients are generally negative, and under all scenarios positive work is performed (i.e., the ecosystems exhibit a net heat loss away from the land surface consistent with the temperature gradient; Fig. 1a). Thus, there is no indication that either of these vegetation scenarios should be considered infeasible since they do not perform negative work persistently over long periods of time.

However, the alpine/Arctic sites with both counterfactual and existing vegetation structures exhibit both positive and negative temperature gradients (i.e., temperature inversions). When no temperature inversion is present, the same trend is apparent as before in which the simulated forests (*red*) at each site perform more work for the corresponding resultant temperature gradient than the existing shrubs/grasses (*blue*). Considering merely the negative temperature gradient (positive x-axis), the simulated forests at the alpine/Arctic sites actually seem more advantageous. However, alpine/Arctic environmental conditions for all three locations have considerable time periods that exhibit temperature inversions and large magnitudes of negative work (i.e the ecosystems exhibit a net heat loss upward in the *opposite* direction of the temperature gradient; Fig. 1b). This large amount of negative work performed indicates a thermodynamic infeasibility through unsustainable heat loss occurring due to the counterfactual trees modeled at the alpine/Arctic sites.

**Figure 4:**
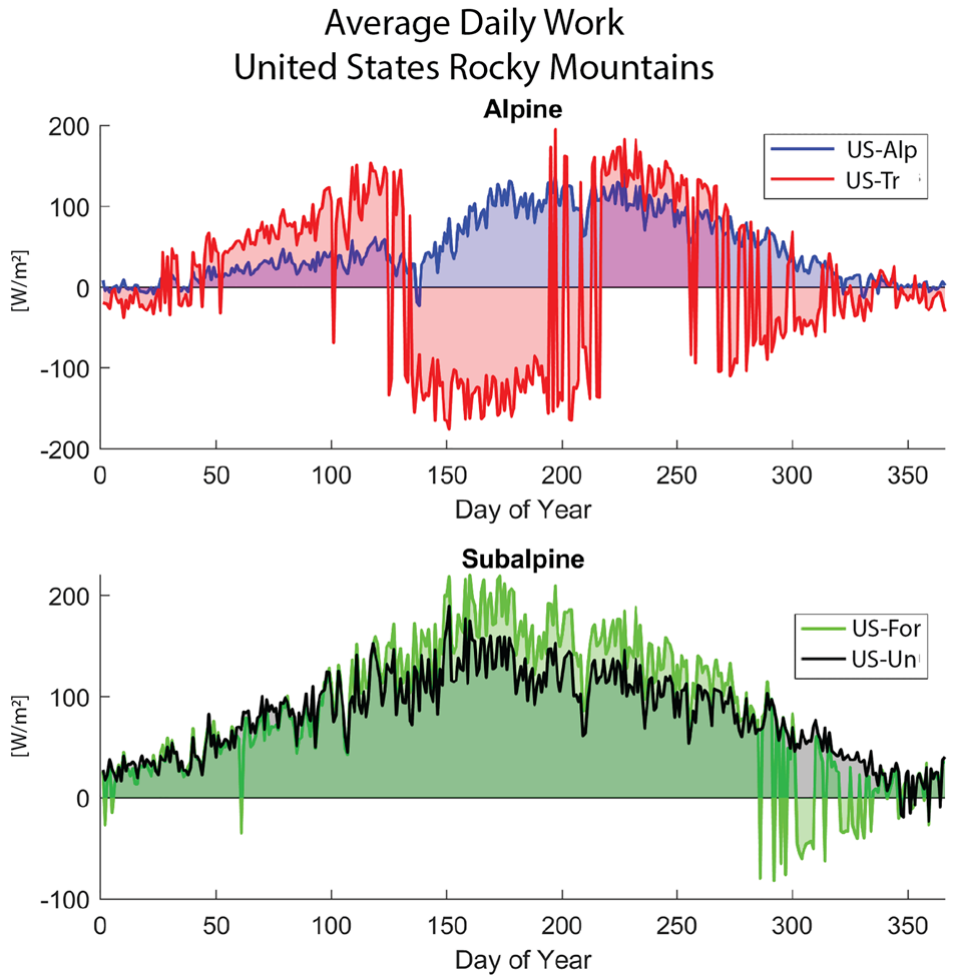
Average daily time series of work for the entire study period (2008–2013) for scenarios in the United States Rocky Mountains. The top panel demonstrates the prolonged negative work (i.e., heat transport in the opposite direction of the temperature gradient) associated with snowmelt and winter temperature inversions of the simulated alpine forest (*red*; US-Tr), indicating that this counterfactual is thermodynamically infeasible. The existing alpine and subalpine vegetation (*blue*, US-Alp & *green*, US-For) generally only experience negative work during snowmelt conditions. The subalpine forest (*green*; US-For) experiences negative work sporadically for short durations during the winter; these instances are a function of snowmelt as well since the snowpack does not persist throughout the winter at this site (see Fig. 5a).

**Figure 5:**
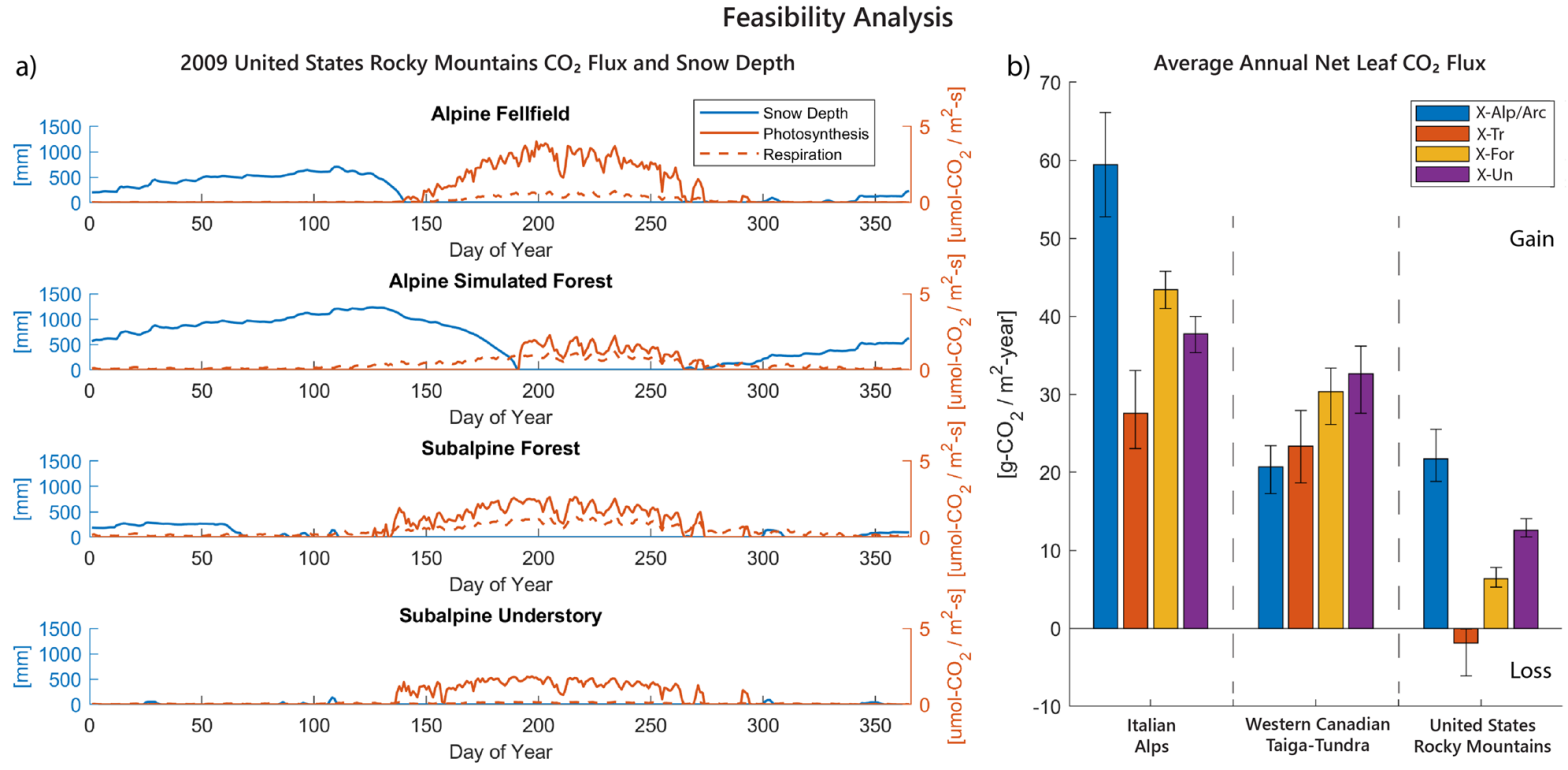
Feasibility analysis demonstrating annual net loss of CO$_2$flux for the United States Rocky Mountains alpine simulated forest counterfactual scenario due to increased snowpack and shortened growing seasons. (**a**) 2009 daily timeseries of modeled snow depth (*blue*) and leaf CO$_2$ flux—the averaged daily photosynthetic CO$_2$ uptake (*orange solid line*) and above-ground autotrophic respiration (*orange dotted line*)—for the United States Rocky Mountains scenarios. For the alpine simulated forest, cooler temperatures near the earth’s surface result in faster accumulation of snow and less snowmelt throughout the winter, extending the time needed to melt the snow. Thus, the simulated forest exhibits compounding snow depth and an abbreviated summer season without snowpack, leading to shortened photosynthesis periods compared to the alpine fellfield scenario (*top panel*). (**b**) Modeled average annual net leaf CO$_2$ flux over the entire study period (IT, 2 years; CA, 3 years; US, 6 years). Positive flux corresponds to net leaf uptake (i.e., photosynthesis minus above-ground autotrophic respiration). Error bars indicate the range of annual net leaf CO$_2$ flux for all years of the study period. Simulated forests for the two alpine sites (*orange*; US-Tr & IT-Tr) experience decreases in net CO$_2$ flux compared to the other scenarios. Further, the US-Tr scenario exhibits overall losses in CO$_2$ year to year, creating unsustainable mass balance for biomass productivity and indicating that the counterfactual is infeasible.

In the three pairs of sites studied, the majority of temperature inversions occur during snowmelt conditions (see Figs. 4 and S5). Snowmelt temperature inversions occur when the air temperature is warmer than the melting snow surface (Fig. 1b). Despite warmer air temperatures, the phase transition associated with snowmelt keeps the surface temperature low. Temperature inversions trap heat within the upper canopy, causing high temperatures in the upper- and mid-canopy layers while layers near the earth surface remain cool due to shading. The shrub/grass (*X-Alp/Arc*), the simulated trees (*X-Tr*), and occasionally the subalpine forest (*X-For*) scenarios exhibit inversions during snowmelt. However, excessive shading from added leaf area, measured as leaf area index (LAI; see Fig. S4), from the simulated trees in the *X-Tr* scenarios extends the snow cover period and causes the snow to take longer to melt and the ecosystem to remain in this inverted state significantly longer, sometimes well into the summer (see Figs. 5a and S6). The result is a net loss of heat upward from the middle and upper layers of the canopy while the lower layers of the control volume at and near the earth surface remain cooler. Thus, heat is lost in the opposite direction of the resultant temperature gradient, corresponding to negative work. Since the LAI of the trees at the sub-Arctic site only reaches around 0.5, the role of tree leaf area on temperature inversions in the CA-Tr scenario is less pronounced, and the duration of temperature inversions is shorter than the US-Tr and IT-Tr scenarios. Alternatively, the existing vegetation scenarios (*X-For*, *X-Un*, and *X-Alp/Arc*) allow for sunlight and heat to penetrate into the lower canopy and warm the melting snow to quickly revert the ecosystem to negative temperature gradients (Fig. 1a).

To demonstrate the prevalence of temperature inversions and negative work performed by the alpine counterfactual forest vegetation, Fig. 4 displays the average daily work for the scenarios in the United States Rocky Mountains. The US-Alp scenario (*blue*) experiences negative work briefly during snowmelt (around day 140). The subalpine forest (*green*; US-For) experiences negative work sporadically for short durations during the winter; these instances are a function of snowmelt as well since the snowpack does not persist throughout the winter at this site (see Fig. 5a). Alternatively, the US-Tr scenario (*red*) experiences long durations of negative work during both snowmelt (approximately days 140-210) and winter conditions (around day 300). The IT-Tr and CA-Tr sites experience similar persistence of negative work behavior during snowmelt (see *Supplementary Material*). The long durations of temperature inversions indicate a thermodynamic infeasibility since ecosystems would not be able to sustain this rate of heat dissipation without energy reaching the lower canopy and soil surface. Based on the analysis of the *X-Tr* scenarios for all three locations, extended periods of unfavorable negative work demonstrates that the existence of trees beyond a treeline would be thermodynamically infeasible.

### Net carbon dioxide loss

In addition to the thermodynamic infeasibility associated with negative work, the extended periods of negative work also lead to another infeasibility. In the alpine counterfactual trees scenarios, these extended periods of negative work lead to excessive net leaf CO$_2$ loss at the US-Tr alpine site and a CO$_2$ disadvantage at the IT-Tr alpine site. The additional shade created by the greater leaf area of the simulated forest vegetation in the alpine environmental conditions of the these two sites leads to an extended snowmelt season with temperature inversions and negative work performed sometimes well into the summer. To illustrate this behavior, Fig. 5a displays the snow depth and average daily photosynthetic and above-ground autotrophic respiration rates obtained from model simulations for each United States Rocky Mountains scenario during 2009. The Italian Alps scenarios exhibit similar (though less extreme) behavior (see Fig. S5a in the *Supplementary Material*). Upon comparing the snow depth trends in the top two panels, the simulated forest ecosystem (US-Tr) accumulates more snow than the alpine fellfield (US-Alp) and has snowpack present well into July, aided by increased shade from the trees. The extended presence of snow in the US-Tr ecosystem prevents photosynthesis from switching on in April or May as it does in the other scenarios^31^. The active photosynthetic period for US-Tr instead begins in July and ends in October, slightly earlier than the other scenarios as well. In contrast, respiration continues to occur throughout the entirety of the summer for all scenarios^32^. Thus, the shorter photosynthetic period leads to an overall net loss in CO$_2$ from the vegetation for the simulated forest scenario.

To demonstrate this CO$_2$ loss on a yearly basis, Fig. 5b displays the total modeled annual net leaf CO$_2$ flux for all scenarios averaged over the years of each location’s study period (see *Supplementary Material*). The Italian Alps counterfactual trees scenario (IT-Tr; *orange*) exhibits a gain of CO$_2$ lower than the other scenarios at this location. Although this may not be an infeasibility, the lower net CO$_2$ gain may not be able to sustain the additional leaf area associated with the trees. Thus, we designate this as a disadvantage for the IT-Tr scenario over the other scenarios in the Italian Alps. Alternatively, the United States Rocky Mountain alpine simulated trees scenario (US-Tr; *orange*) exhibits a net loss of CO$_2$. This annual net loss of CO$_2$ indicates that the existence of trees under alpine environmental conditions is not sustainable in the long term. Thus, the existence of the forest vegetation at the alpine site should be considered infeasible. Further, this CO$_2$ loss is a result of a feedback loop initiated by the increased leaf area of the simulated trees. The excess shade slows the melting of the snow. By the time the snow fully melts, the delayed start of photosynthetic CO$_2$ uptake results in a shortened photosynthesis window such that the overall gain of CO$_2$ is unable to account for the loss of CO$_2$ from respiration. For ecosystems to exist, their leaf CO$_2$ exchange (i.e., photosynthetic CO$_2$ uptake minus above-ground autotrophic respiration) must be positive at minimum. Thus, annual net CO$_2$ loss indicates another infeasibility. Although this infeasibility manifests through excessive CO$_2$ loss, it is in fact also a consequence of changes in thermodynamic behavior.

### Thermodynamic limits

**Figure 6:**
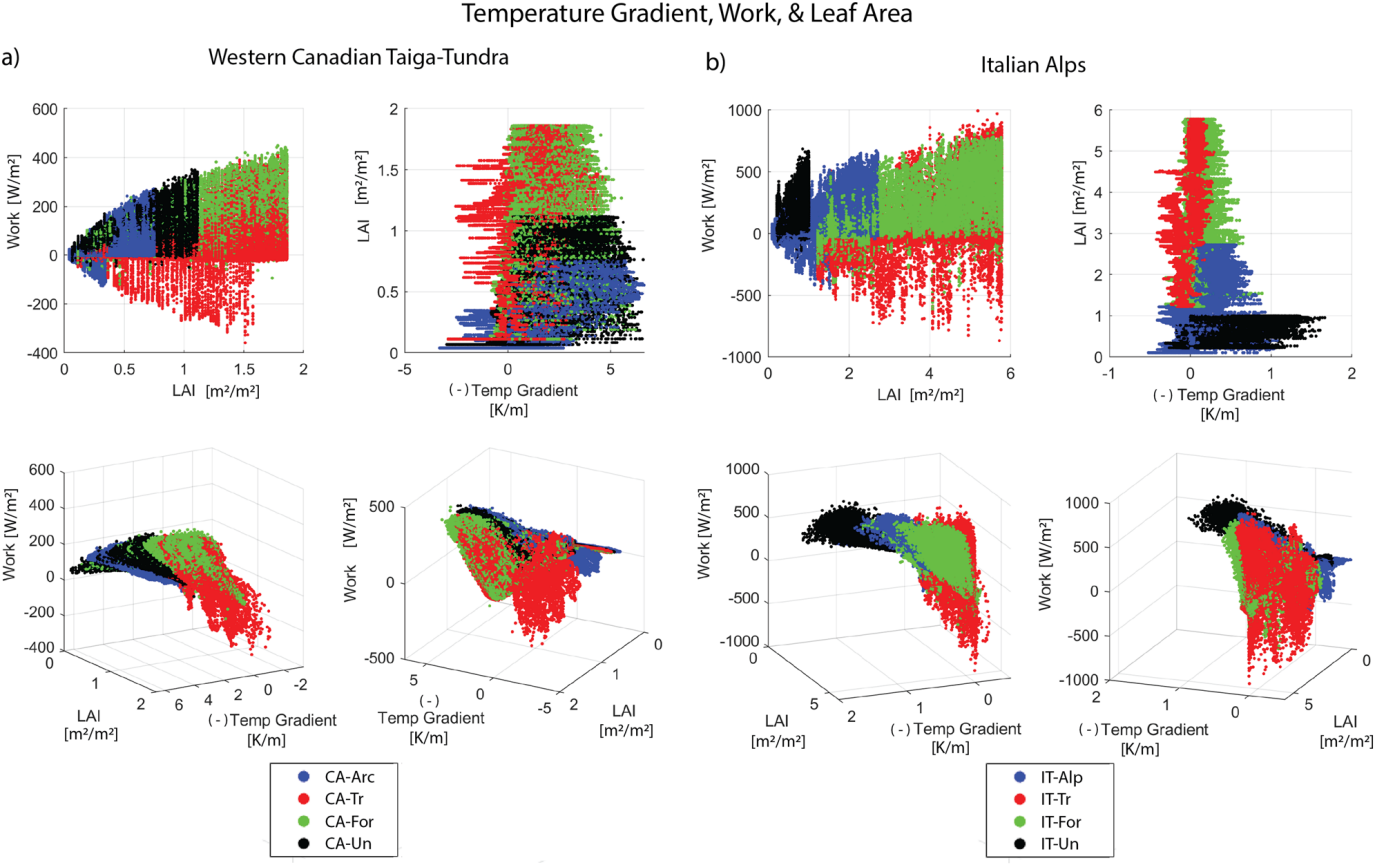
Four projected views from the 3-D plot of work, temperature gradient, and total leaf area index (LAI) for the (**a**) Western Canadian Taiga-Tundra and (**b**) Italian Alps scenarios. The top panels display that increases in LAI lead to increases in the magnitude of work and smaller resultant temperature gradients. The negative of the resultant temperature gradient is plotted. Thus, positive values refer to negative temperature gradients such that larger values indicate stronger declines in temperature from the earth surface to the atmosphere. Negative values indicate positive temperature gradients, or temperature inversions. The 3-D views in the bottom panels show the transition from flatter curves to greater magnitudes of work with increases in resultant temperature gradient as more LAI is incorporated for each set of environmental conditions (i.e., alpine, subalpine). The simulated alpine/Arctic forest scenarios (*red*; *X-Tr*) exhibit considerable negative work performed since the LAI is beyond the supported limit of the local environmental conditions.

The analyses described in the previous sections demonstrate how the alpine counterfactual tree scenarios lead to thermodynamic infeasibilites expressed through extensive negative work performed and annual net CO$_2$ loss. To better understand the role of additional leaf area resulting from the incorporation of trees in the counterfactual alpine/Arctic scenarios, total LAI is plotted alongside work and resultant temperature gradient (from Fig. 3) for the Western Canadian Taiga-Tundra (Fig. 6a) and the Italian Alps (Fig. 6b). The top-left panel for both locations demonstrates increasing magnitudes of work, both positive and negative, as LAI increases. In the top-right panel, the simulated trees scenarios (red; *X-Tr*) have resultant temperature gradients hovering closer to zero in comparison to the other three scenarios across the entire range of annual LAI, indicating that this vegetation scenario is more effective at dissipating heat throughout the year. The bottom panels show that the marginal work performed for a negative temperature gradient is greatest for Arctic/alpine simulated tree scenarios (red; *X-Tr*). However, the work performed under a positive temperature gradient is largely negative due to the continued loss of heat from the ecosystem (against the temperature gradient) during temperature inversions. The existing Arctic/alpine scenarios (blue; *X-Arc/Alp*) exhibit negative work fluxes as well, but with much lower magnitude. These two plots further demonstrate that as LAI increases, the work versus temperature gradient relationship transitions from lower to higher magnitudes of work, yielding significant negative work performed by the counterfactual forest vegetation structure (*X-Tr*), which demonstrates thermodynamic infeasibility.

We use the Western Canadian Taiga-Tundra and the Italian Alps as examples here to demonstrate that the infeasibility conditions do not need to manifest in terms of net CO$_2$ loss for the counterfactual tree scenario (*X-Tr*) to become infeasible. The thermodynamic infeasibility associated with prolonged negative work performance alone is enough to prevent the existence of trees at the Arctic site. For additional context, the sub-Arctic site is located within the forest-tundra ecotone, so the prevalence of trees is less dense than sites further below the treeline. Because of this, the difference in leaf area is not as great between CA-Tr and CA-Arc scenarios as the other sites. Thus, the snowmelt is not excessively prolonged, and the photosynthesis window does not shorten substantially. Thus, the Arctic simulated trees scenario (CA-Tr) does not exhibit the infeasibility or disadvantage from CO$_2$ loss in comparison with the other two regions. Even so, trees do not exist on the Arctic site, and we attribute this to the thermodynamic infeasibility instituted from the negative work and temperature inversions from the extended snowmelt and snow cover season.

Overall, the results highlighted in Fig. 6 demonstrate that marginal changes in work have a positive relationship with LAI, yielding considerably larger magnitudes of work (both positive and negative) with increases in LAI. Further, the persistence of resultant temperature gradients for the Arctic/alpine counterfactual trees scenarios (*X-Tr*) around and below zero throughout the year indicates that the greater dissipation of heat by this vegetation structure leads to feedbacks such that the resultant temperature gradient inverts and negative work becomes common. Thus, the vegetation for the *X-Tr* counterfactual scenarios is too effective at dissipating heat for the given incoming radiation and environmental conditions. This behavior is consistent for all three locations considered in this study (see Fig. S7 in the *Supplementary Material*). Based on these observations, we conclude that the negative work demonstrated during temperature inversions in the simulated forest scenarios (*X-Tr*) offsets the advantages (greater marginal work increases) exhibited during negative resultant temperature gradients. Thus, for each of the three locations studied, trees modeled beyond the treeline are thermodynamically infeasible.

## Discussion

The counterfactual scenarios of trees simulated at alpine and Arctic sites resulted in thermodynamic conditions of infeasibility at all three locations modeled in this study: the Italian Alps, the United States Rocky Mountains, and the Western Canadian Taiga-Tundra. Further, the results from all three locations demonstrate how the relationship among LAI, work, and temperature gradient reveals that the additional leaf area associated with trees at Arctic/alpine sites leads to higher magnitudes of work throughout the year, forcing a positive resulting temperature gradient. We, therefore, discuss and propose a new framework associated with this phenomenon in terms of the thermodynamic behavior of ecosystems.

As previously mentioned, existence of vegetation is made thermodynamically feasible through the establishment of a temperature gradient from the earth surface to the air above the canopy that supports positive work associated with the net dissipation of heat. Based on the results presented, we propose that the vegetation and associated structural properties such as leaf area present in an ecosystem are directly related to the strength of the incoming radiation and its feedback with local environmental conditions, which we refer to as a location’s *potential dissipation capacity*.

**Figure 7:**
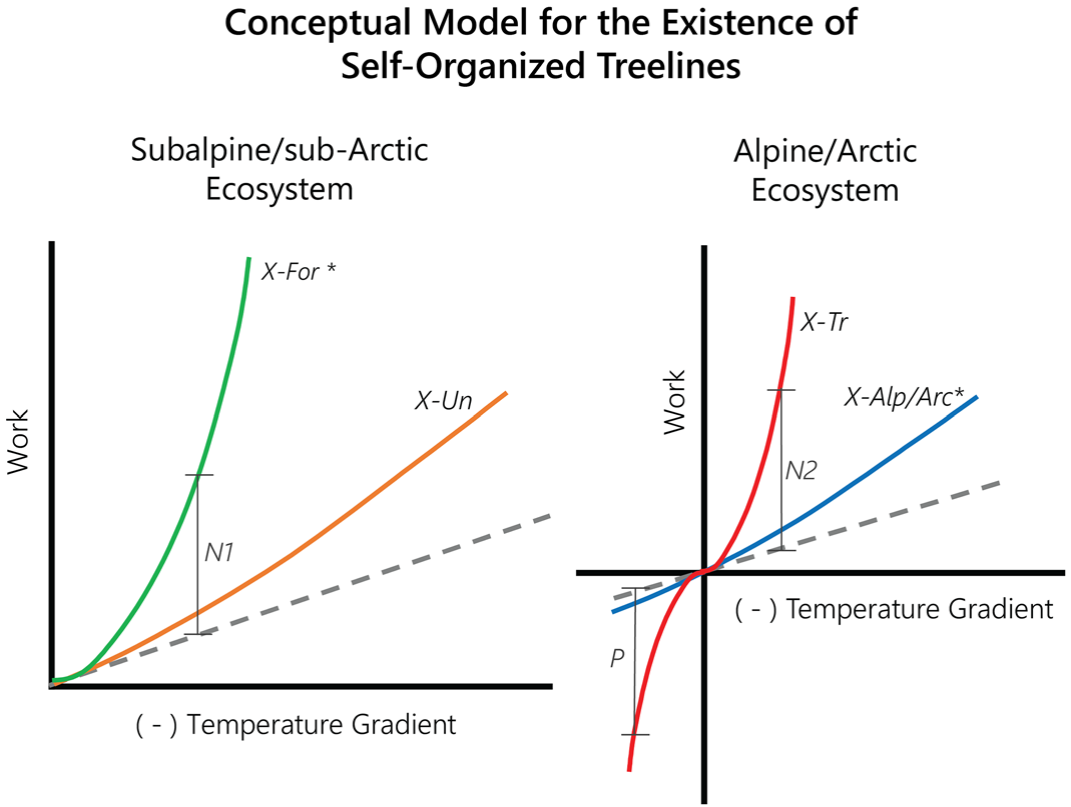
Conceptual model for the existence of treelines as a result of self-organization of the thermal environment. Self-organized treelines are demonstrated through the balance of positive or negative feedback between the resulting temperature gradient and the work performed by various vegetation structures. The negative of the resultant temperature gradient is plotted on the x-axis. Thus, the positive x-axis refers to a negative temperature gradient such that larger values indicate stronger declines in temperature from the earth surface to the atmosphere. The negative x-axis indicates positive temperature gradients, or temperature inversions. The dotted line represents an ecosystem without vegetation (i.e., bare soil). The colored solid lines represent vegetation curves as defined in Table 1. Dissipation rates leading to negative (*N1* or *N2*) or positive (*P*) feedback loops between temperature gradient and vegetation structure are shown as the vertical distance from the bare soil curve to the vegetation scenarios. The starred scenario on each plot represents the most advantageous viable vegetation structure for the given ecosystem. The plot on the left represents ecosystems in which both vegetation scenarios (*X-For* & *X-Un*) are viable options, and the *X-For* scenario is most advantageous. The plot on the right represents ecosystems in which one of the vegetation scenarios (*X-Tr*) is infeasible due to positive feedback loops that result in continued dissipation of heat during temperature inversions. Instead, the *X-Alp/Arc* scenario is the most advantageous viable vegetation structure.

Similar to the concept of potential evapotranspiration or potential net ecosystem production^33^, potential dissipation capacity indicates the maximum possible dissipation of heat that is thermodynamically feasible in a given ecosystem. In ecosystems, vegetation structure and temperature gradient self-organize concurrently based on the potential dissipation capacity of a given ecosystem. The resultant temperature gradient is a consequence of the net radiation, air temperature and other environmental conditions, and the dissipation of heat performed by the vegetation itself, or the actual dissipation rate. This process can be represented by either a positive or negative feedback loop (Fig. 7). Feasible vegetation structures perform work equivalent to or below their ecosystem’s potential dissipation capacity, meaning heat dissipation and positive work lead to lower surface temperatures and weaker resultant temperature gradients, indicating a negative feedback loop. When the leaf area present dissipates heat beyond the potential dissipation capacity of an ecosystem (e.g., simulated trees at an alpine/Arctic site), the resultant temperature gradient becomes positive, leading to prolonged temperature inversions and thermodynamic infeasibility. In this case, a positive feedback loop occurs such that additional heat dissipation further inverts the temperature gradient leading to additional negative work performed. With a lower potential dissipation capacity, less leaf area is needed to dissipate heat throughout the ecosystem on an annual basis, and the ecosystem will self-organize towards a less effective dissipative structure (e.g., shrubs or grasses only) in response. Additional leaf area then becomes less advantageous, or even infeasible. The inherent thermodynamic difference above and below the treeline is the following: the potential dissipation capacity of the ecosystem is greater below the treeline than above, resulting in the need for more effective dissipators, or trees. Trees and their additional leaf area are too productive at performing work for the sites beyond the treeline, and thus, they do not exist.

Figure 7 provides a conceptual model representing the thermodynamic behavior of the subalpine/sub-Arctic and alpine/Arctic ecosystems. Both plots present three curves representing different vegetation scenarios for a given ecosystem with set environmental conditions: bare soil (*dotted line*), understory only (*orange, blue*), and forest vegetation including understory and overstory trees (*green, red*). The vertical distance from the dotted line to any vegetation scenario curve at a given temperature gradient indicates the rate of work performed as a result of self-organized vegetation structure. When the vegetation curve is above the dotted line, then the vertical distance (*N1* or *N2*) represents the energy dissipation that results in a typical negative feedback loop between the vegetation and the temperature gradient. When the vegetation curve is below the dotted line (*X-Tr*), then the vertical distance (*P*) represents the energy dissipation that results in a positive feedback loop between vegetation and the temperature gradient, meaning that the ecosystem loses heat against the resultant temperature gradient. When strong enough, this positive feedback results in prolonged temperature inversions and thermodynamic infeasibility. The subalpine/sub-Arctic ecosystem diagram on the left represents ecosystems in which all vegetation scenarios are viable options, and the *X-For* scenario is most advantageous. The alpine/Arctic ecosystem diagram on the right represents ecosystems in which one of the vegetation scenarios (*X-Tr*) is infeasible due to a strong positive feedback loop that results in continued dissipation during temperature inversions. As a result, the *X-Alp/Arc* scenario is the most advantageous viable vegetation structure.

*N1* and *N2* in Fig. 7 indicate the additional work performed from dissipative structures (i.e., vegetation) for a given resultant temperature gradient. This represents the improved ability of the ecosystem to export energy from the earth surface to the air above the canopy rapidly and efficiently. However, this paper demonstrates that more dissipation does not always indicate the optimal or more probable state, such as in the case of trees simulated beyond a treeline. In this case, the trees actually transport energy out of the ecosystem more quickly than warranted by the ecosystem’s potential dissipation capacity, resulting in considerable temperature inversions, as demonstrated by *P* in Fig. 7. The incoming radiation and environmental conditions of the alpine and Arctic sites do not yield a strong enough potential dissipation capacity to warrant the dissipation of heat facilitated by the *X-Tr* vegetation; thus, prolonged temperature inversions occur. Overall, trees cause heat dissipation that is not needed or beneficial to the ecosystem under alpine or Arctic conditions.

This research opens the door for further study defining the naturally-occurring transition of vegetation structures. We present potential dissipation capacity as a theoretical concept for understanding the maximum dissipation rate for any given ecosystem, dependent on the incoming radiation and environmental conditions at its location. We anticipate that there is a relation among incoming radiation, temperature, and other local properties, such as emissivity and reflectance, that could further define this concept into a measurable quantity. The definition of such a relation would enable scientists to anticipate changes in vegetation structure from human-induced perturbations as well as gradual alterations to environmental conditions.

### Conclusion

Based on the results of this study, we conclude that the trees do not exist beyond a treeline because they would be thermodynamically infeasible due to the considerable negative work as demonstrated by the counterfactual scenarios for all Arctic/alpine locations, *X-Tr*. An additional condition of thermodynamic infeasibility limits trees from existing beyond the treeline due to the annual net loss of CO$_2$ resulting from compounding accumulation of snowpack and shortened photosynthesis windows exhibited at the United States Rocky Mountains counterfactual alpine scenario, US-Tr. Thus, thermodynamic infeasibility, whether due to negative work or annual net CO$_2$ loss, demonstrates the utility of the thermodynamic approach beyond what can be determined from a mechanistic approach (e.g., temperature limitation or net CO$_2$ loss).

Overall, the two conditions of infeasibility associated with this counterfactual elucidate the thermodynamic requirement for the existence of vegetation structure. The thermodynamic basis for the demarcation of Arctic and alpine treelines is determined by the location beyond which the dissipation rate of trees is greater than what is needed for the ecosystem based on feedback loops among the local environmental conditions and the concurrent self-organization of vegetation structure and temperature gradient, or its potential dissipation capacity.

This research arrives at a thermodynamic theory for the directionality of ecosystem self-organization towards the vegetation structure that most effectively dissipates heat without resulting in a positive feedback loop leading to prolonged temperature inversions. This dissipation rate will correlate to the magnitude of the potential dissipation capacity at a given site. Therefore, greater potential dissipation capacity calls for higher dissipation rates enabled by multiple functional groups and increased LAI. When such vegetation structure with multiple functional groups produces higher dissipation rates than the ecosystem’s potential dissipation capacity—as is the case for forest vegetation with trees beyond a treeline—the vegetation structure is then thermodynamically infeasible and does not exist.

These findings provide a thermodynamic explanation for the demarcation of treelines at specific point in time. However, as described in the Introduction, there are entire bodies of work looking into the physiological and resource limitations that prevent the existence of trees during certain conditions. Those studies describe the physical characteristics of vegetation self-organization that determine the actual dissipation rate of an ecosystem at a given location. Alternatively, the dissipation capacity of an ecosystem is based on the energy availability and environmental conditions at that location regardless of present vegetation. Thus, the fundamental condition of thermodynamic feasibility is simultaneously a consequence of energy availability and local environmental conditions and a control on the physical vegetation that can exist. Thermodynamic feasibility sets bounds for the structure of vegetation that is able to self-organize; the diverse physical limitations that manifest (e.g., maximum stem height, seedling dispersal, etc.) exhibit the influence of the historic evolution of the treeline and local vegetation. Therefore, the thermodynamic theory as described in this paper is complementary to the existing literature investigating the biotic and abiotic factors that lead toward the demarcation of treelines.

## Methods

### Experimental design

The multi-layer canopy model, MLCan, models the mass and energy fluxes occurring within the canopy, roots, and soil system to resolve the energy and entropy fluxes, and associated work efficiencies for each ecosystem^12,20–23^. Observed environmental data—including wind speed, air temperature, global radiation, precipitation, friction velocity, air pressure, and relative humidity—from various sources^34–39^ were used as forcing for the model. Table S1 of the *Supplementary Material* documents additional model parameters for MLCan for all sites. Model outputs include soil and canopy layer temperatures, snow depth, photosynthesis and respiration rates, and energy and entropy fluxes at each timestep.

For all sites, the model was run on a half-hourly time scale. The study period for each pair of sites above and below the treeline was chosen as the longest consecutive time series of available data for both sites: 2012-2013 for the Italian Alps, 2008-2013 for the United States Rocky Mountains, and 2016-2018 for the Western Canadian Taiga-Tundra. MLCan has been validated for numerous sites across the Western Hemisphere^12,20–26^. To apply MLCan to the harsh winter conditions of Arctic and alpine ecosystems, we included new parameterizations for the hydraulic and thermal properties of peat soils and photosynthesis switches for dormancy during winter. Additional information on model updates and site-specific validation, parameterization, and pre-processing methods can be found in the *Supplementary Material*.

### Site descriptions

The eddy covariance data for the IT sites are taken from the Lavarone (Lav) and Monte Bondone (MBo) sites in the FLUXNET2015 network^34,35^, located in the Trento province in the Eastern Italian Alps, with a 200 m elevation difference. The LAV site is an evergreen needle-leaf subalpine forest at 1300 m above mean sea level (MSL) (45.95620$^{\circ }$ N, 11.28132$^{\circ }$ E), consisting of predominantly European silver fir (*Abies alba* Mill.) with a suppressed beech (*Fagus sylvatica* L.) understory^40^. The MBO site is an alpine meadow located on a mountain karst plateau at 1500 m above MSL (Viote del Monte Bondone; 46.01468$^{\circ }$ N, 11.04583$^{\circ }$ E), dominated by perennial bunchgrass (*Nardus stricta* L.)^41,42^.

The eddy covariance data for the US sites are taken from the Niwot Ridge (NR1) site in the AmeriFlux network^38^ and the “T-Van” location in Knowles et al.^39,43^, located 5 km apart in distance with a 430 m elevation difference near the Continental Divide in the United States Rocky Mountains in Colorado. The NR1 site (40.0329$^\circ$ N, 105.5464$^\circ$ W; 3050 m elevation) is an evergreen needleleaf forest dominated by subalpine fir (*Abies lasiocarpa* var. *bifolia*) and Englemann spruce (*Picea engelmannii*) with sparse understory vegetation comprised of wild blueberry (*Vaccinium myrtillus*)^44–46^. The T-Van site (40.05305$^\circ$N; 105.58639$^\circ$W; 3480m above MSL) is an alpine fellfield consisting of curly sedge (*Carex rupestris*)^39,47^.

The eddy covariance data for the CA sites are taken from the Havikpak Creek (HPC) and Trail Valley Creek (TVC) AmeriFlux sites^36,37^, located 50 km apart on either side of the Arctic treeline in Northwest Territories along the Western Canadian Taiga-Tundra interface. The sites consist of peat soil above mineral soil and continuous permafrost^48–51^. HPC (68.32029$^{\circ }$ N, 133.51878$^{\circ }$ W; 80 m above MSL) exists in the forest-tundra ecotone in the Taiga. It is a sparse needleleaf boreal forest dominated by black spruce (*Picea mariana*) with small shrub species (*Ledum sp.*, *Ledum groenlandicum*) at 80 m elevation^52^. TVC (68.7462$^{\circ }$ N, 133.5017$^{\circ }$ W; 85 m above MSL) is an Arctic tundra site consisting of short grasses and berry species (*Ledum groenlandicum*)^49,50,53^.

Additional information for all sites, including LAI and data pre-processing, can be found in the *Supplementary Material*.

### Thermodynamic calculations

Temperature gradient and work are key concepts in our framework for understanding thermodynamic behavior of the self-organized ecosystem structure above and below the treeline. For our study we calculate the resultant temperature gradient, arising as a result of a self-regulated thermal environment in the canopy, as:

1 $$\begin{aligned} \frac{\Delta T}{\Delta z} = \frac{T_{air}-T_{surf}}{h_e} \end{aligned}$$

where $T_{surf}$ and $T_{air}$ are the temperature (in Kelvin) of the soil surface and the air above the canopy, respectively, and $h_e$ is the ecosystem height, taken as the maximum canopy height in each site pair (see Table S1 in the *Supplementary Material*)^12^.

In our 1-D model simulations, heat fluxes are assumed positive in the positive *z* direction, leaving the ecosystem control volume into the atmosphere above the canopy. However, work is defined based on the direction of the temperature gradient. When the work performed by the ecosystem, as measured by the sum of latent and sensible heat, is consistent with the temperature gradient it is designated as positive work, otherwise as negative work. To demonstrate this relationship, work is augmented from a prior formulation^12^ as follows:

2 $$\begin{aligned} Work = (LE+H) \times - sign(\Delta T) \end{aligned}$$

in which work, latent heat (*LE*), and sensible heat (*H*) are in units of W/m$^2$, and $\Delta T = T_{air}-T_{surf}$. Heat leaving the bottom of the control volume due to water loss is negligible, and thus ignored.

## Supplementary Information

Supplementary Information.

## Data Availability

The MLCan model code is available in the HydroComplexity GitHub: https://github.com/HydroComplexity/MLCan4.0.

## References

[1] Richardson AD, Friedland AJ (2009). A review of the theories to explain arctic and alpine treelines around the world. J. Sustain. For..

[2] Callaghan, T. V., Werkman, B. R. & Crawford, R. M. M. The tundra-taiga interface and its dynamics: concepts and applications. Ambio. Special Report Number 12, 6–14. http://www.jstor.org/stable/25094570 (Springer, 2002). 12374061

[3] Holtmeier F-K, Broll G (2020). Treeline research-from the roots of the past to present time. A review. Forests.

[4] Wardle P (1971). An explanation for alpine timberline. N. Z. J. Bot..

[5] Shi P, Körner C, Hoch G (2008). A test of the growth-limitation theory for alpine tree line formation in evergreen and deciduous taxa of the eastern Himalayas. Funct. Ecol..

[6] Gartner, B. L. *Plant Stems: Physiology and Functional Morphology* (Elsevier, 1995).

[7] Körner, C. *Alpine Plant Life: Functional Plant Ecology of High Mountain Ecosystems* (Springer Nature, 1999).

[8] Harsch MA, Hulme PE, McGlone MS, Duncan RP (2009). Are treelines advancing? A global meta-analysis of treeline response to climate warming. Ecol. Lett..

[9] Kauffman, S. A. *et al.**The Origins of Order: Self-Organization and Selection in Evolution* (Oxford University Press, 1993).

[10] Camazine, S. *et al.**Self-Organization in Biological Systems* (Princeton University Press, 2003).

[11] Lavorel S, McIntyre S, Landsberg J, Forbes T (1997). Plant functional classifications: from general groups to specific groups based on response to disturbance. Trends Ecol. Evolut..

[12] Richardson M, Kumar P (2020). Discerning the thermodynamic feasibility of the spontaneous coexistence of multiple functional vegetation groups. Nat. Sci. Rep..

[13] Schneider ED, Kay JJ (1994). Life as a manifestation of the second law of thermodynamics. Math. Comput. Model..

[14] Schneider ED, Kay JJ (1994). Complexity and thermodynamics: towards a new ecology. Futures.

[15] Kondepudi, D. & Prigogine, I. *Modern Thermodynamics: From Heat Engines to Dissipative Structures* (John Wiley & Sons, 2014).

[16] Walsh SJ, Butler DR, Allen TR, Malanson GP (1994). Influence of snow patterns and snow avalanches on the alpine treeline ecotone. J. Veg. Sci..

[17] Nauta AL (2015). Permafrost collapse after shrub removal shifts tundra ecosystem to a methane source. Nat. Clim. Change.

[18] Whiteman, C. D. *Mountain Meteorology: Fundamentals and Applications* (Oxford University Press, 2000).

[19] Kahl JD, Serreze MC, Schnell RC (1992). Tropospheric low-level temperature inversions in the Canadian arctic. Atmos. Ocean.

[20] Drewry D (2010). Ecohydrological responses of dense canopies to environmental variability: 1. Interplay between vertical structure and photosynthetic pathway. J. Geophys. Res. Biogeosci..

[21] Le PV, Kumar P, Drewry DT (2011). Implications for the hydrologic cycle under climate change due to the expansion of bioenergy crops in the Midwestern United States. Proc. Natl. Acad. Sci..

[22] Le PV, Kumar P, Drewry DT, Quijano JC (2012). A graphical user interface for numerical modeling of acclimation responses of vegetation to climate change. Comput. Geosci..

[23] Quijano JC, Kumar P, Drewry DT, Goldstein A, Misson L (2012). Competitive and mutualistic dependencies in multispecies vegetation dynamics enabled by hydraulic redistribution. Water Resour. Res..

[24] Quijano JC, Kumar P, Drewry DT (2013). Passive regulation of soil biogeochemical cycling by root water transport. Water Resour. Res..

[25] Quijano JC, Kumar P (2015). Numerical simulations of hydraulic redistribution across climates: the role of the root hydraulic conductivities. Water Resour. Res..

[26] Lee E (2018). Impact of hydraulic redistribution on multispecies vegetation water use in a semiarid savanna ecosystem: an experimental and modeling synthesis. Water Resour. Res..

[27] Domingues TF, Martinelli LA, Ehleringer JR (2007). Ecophysiological traits of plant functional groups in forest and pasture ecosystems from eastern Amazonia, Brazil. Plant Ecol..

[28] Bonan GB, Levis S, Kergoat L, Oleson KW (2002). Landscapes as patches of plant functional types: an integrating concept for climate and ecosystem models. Glob. Biogeochem. Cycles.

[29] Wullschleger SD (2014). Plant functional types in earth system models: past experiences and future directions for application of dynamic vegetation models in high-latitude ecosystems. Ann. Bot..

[30] Ustin SL, Gamon JA (2010). Remote sensing of plant functional types. New Phytol..

[31] Knowles JF, Burns SP, Blanken PD, Monson RK (2015). Fluxes of energy, water, and carbon dioxide from mountain ecosystems at Niwot Ridge, Colorado. Plant Ecol. Divers..

[32] Grogan P (2012). Cold season respiration across a low arctic landscape: the influence of vegetation type, snow depth, and interannual climatic variation. Arct. Antarct. Alp. Res..

[33] Peichl M (2013). Convergence of potential net ecosystem production among contrasting C3 grasslands. Ecol. Lett..

[34] Gianelle, D., Cavagna, M., Zampedri, R. & Marcolla, B. FLUXNET2015 IT-MBo Monte Bondone. 10.18140/FLX/1440170 (2016).

[35] Gianelle, D., Zampedri, R., Cavagna, M. & Sottocornola, M. FLUXNET2015 IT-Lav Lavarone, Dataset. 10.18140/FLX/1440169 (2003–2014).

[36] Sonnentag, O. & Marsh, P. AmeriFlux CA-HPC Havikpak Creek, Ver. 1-5, AmeriFlux AMP, (Dataset). 10.17190/AMF/1773392 (2021).

[37] Sonnentag, O. & Marsh, P. AmeriFlux CA-TVC Trail Valley Creek, Ver. 1-5, AmeriFlux AMP, (Dataset). 10.17190/AMF/1767831 (2021).

[38] Blanken, P. D., Monson, R. K., Burns, S. P., Bowling, D. R. & Turnipseed, A. A. AmeriFlux US-NR1 Niwot Ridge Forest (LTER NWT1), Dataset. 10.17190/AMF/1246088 (1998-).

[39] Knowles JF, Blanken PD, Williams MW, Chowanski KM (2012). Energy and surface moisture seasonally limit evaporation and sublimation from snow-free alpine tundra. Agric. For. Meteorol..

[40] Marcolla B, Pitacco A, Cescatti A (2003). Canopy architecture and turbulence structure in a coniferous forest. Bound. Layer Meteorol..

[41] Papale D (2015). Carbon, Water and Energy Fluxes of Terrestrial Ecosystems in Italy.

[42] Tudoroiu M (2016). Negative elevation-dependent warming trend in the Eastern Alps. Environ. Res. Lett..

[43] Knowles, J. Infilled climate and heat flux data for Tvan towers data loggers (CR3000), 2008—ongoing. ver 1. Environmental Data Initiative., 10.6073/pasta/10fb65e51cd04631bb80c82288b5c51a (2018).

[44] Bowling DR (2018). Limitations to winter and spring photosynthesis of a Rocky Mountain subalpine forest. Agric. For. Meteorol..

[45] Burns S, Blanken P, Turnipseed A, Hu J, Monson R (2015). The influence of warm-season precipitation on the diel cycle of the surface energy balance and carbon dioxide at a Colorado subalpine forest site. Biogeosciences.

[46] Turnipseed A, Blanken P, Anderson D, Monson RK (2002). Energy budget above a high-elevation subalpine forest in complex topography. Agric. For. Meteorol..

[47] Blanken PD (2009). A comparison of water and carbon dioxide exchange at a windy alpine tundra and subalpine forest site near Niwot Ridge, Colorado. Biogeochemistry.

[48] Krogh SA, Pomeroy JW, Marsh P (2017). Diagnosis of the hydrology of a small Arctic basin at the tundra-taiga transition using a physically based hydrological model. J. Hydrol..

[49] Wilcox EJ (2019). Tundra shrub expansion may amplify permafrost thaw by advancing snowmelt timing. Arct. Sci..

[50] Wallace CA, Baltzer JL (2020). Tall shrubs mediate abiotic conditions and plant communities at the taiga-tundra ecotone. Ecosystems.

[51] Street LE (2016). Redox dynamics in the active layer of an arctic headwater catchment; Examining the potential for transfer of dissolved methane from soils to stream water. J. Geophys. Res. Biogeosci..

[52] Helbig M (2016). Addressing a systematic bias in carbon dioxide flux measurements with the EC150 and the IRGASON open-path gas analyzers. Agric. For. Meteorol..

[53] Eaton AK, Rouse WR, Lafleur PM, Marsh P, Blanken PD (2001). Surface energy balance of the western and central Canadian subarctic: variations in the energy balance among five major terrain types. J. Clim..

