# Supplementary material for: Thermodynamic basis for the demarcation of Arctic and alpine treelines
Source: Sci Rep. 2022 Jul 22;12:12565. doi: 10.1038/s41598-022-16462-2 (PMC9307831; doi:10.1038/s41598-022-16462-2)
Supplement: Supplementary file 1 — Supplementary Information. [file 41598_2022_16462_MOESM1_ESM.pdf]

Thermodynamic basis for the demarcation of Arctic and alpine tree-line

Meredith Richardson Martin, Praveen Kumar, Oliver Sonnentag, and Philip Marsh

Supplementary Materials

Supplementary Text
Table S1
Figs. S1 to S7

Supplementary Text

Model Inputs & Pre-processing

Italian Alps. Data for the Italian Alps sites were taken from the FLUXNET2015 Lavarone (Lav) and Monte Bondone (MBo) sites[1,2] and gap-filled using the REddyProc online tool[3]. Validation for the Lav and MBo sites is illustrated in Figure S1.

Western Canadian Taiga-Tundra. The Western Canadian Taiga-Tundra site pair was composed of the Havikpak Creek (HPC) and Trail Valley Creek (TVC) AmeriFlux sites[4,5]. Precipitation data was available at the daily timescale. This was disaggregated to a half-hourly timestep for model simulation using the Bartlett-Lewis rectangular pulse method[6]. There were many gaps in incoming shortwave radiation for the HPC site. These gaps were filled by linear regression with no intercept based on available observed data pairs with the TVC site. Validation for the HPC and TVC sites is illustrated in Figure S2.

United States Rocky Mountains. For the United States Rocky Mountains in Colorado, the alpine data is taken as the T-Van site from Knowles et al.[7], and the subalpine data is taken from the Niwot Ridge (NR1) site in the AmeriFlux network[8,9]. Incoming shortwave radiation was taken from the Subnivean lab site 500m from the T-Van site. The precipitation data is hourly data from the the nearby Saddle site[10] that was disaggregated from hourly to evenly-distributed half-hourly data. The NR1 site has subalpine firs (*Abies lasiocarpa* var. *bifolia*) and Englemann spruce (*Picea engelmannii*) west of the tower, and lodgepole pine (*Pinus contorta*) east of the tower[11–13]. We use the species located west of the tower as the dominant vegetation in our simulations due to the direction of the prevailing winds[12]. Validation for NR1 and T-Van is illustrated in Figure S3.

Leaf Area Index. Time-series of leaf area index (LAI) for all sites (Fig. S4) were interpolated from MODIS[14–20] and calibrated and partitioned as in Richardson and Kumar[21] based on site documentation: Lav – Marcolla and Cescatti, 2003[22] and Lemoine et al, 2002[23], MBo – Gianelle et al, 2009[24], NR1 – Turnipseed et al, 2002[13] and Knowles et al, 2015[25], T-Van – Knowles et al, 2012[7] and Blanken et al, 2009[26], HPC & TVC – Krogh et al, 2017[27] and site measurements. HPC and TVC curves were scaled to fit in-situ observations. LAI (LAI-2200 Plant Canopy Analyser, Li-COR Biosciences, Lincoln, NE) at HPC and TVC were estimated using methods outlined in Ryu et al, 2010[28] and Sonnentag et al, 2007a and 2007b[29,30]. For the HPC site on September 3, 2018, tree LAI was measured at 0.34 ± 0.16 (n = 39) and shrub LAI was 0.51 ± 0.19 (n=39). We assumed a constant partitioning percentage between trees and shrubs within HPC based on the observed scale. For the TVC site on September 1, 2018, LAI was measured as 0.38 ± 0.26 (n = 27). Fifth degree polynomials were fitted to all forest LAI and scaled to include the peaks. All alpine/tundra sites were fitted with fifth degree (MBo & TVC) or fourth degree (T-Van) curves during the time periods with vegetation present. During winter, minimum LAI is assumed to be 0.1 – the minimum detectable LAI in MODIS. Data gaps in the winter for the Western Canadian Taiga-Tundra sites were also assumed to be 0.1. Leaf area density profiles were taken from literature[22,31,32] and fitted to Weibull distributions[33,34].

MLCan Model Updates

MLCan has been validated for numerous sites across the Western Hemisphere[21,35–41]. To apply MLCan to the harsh winter conditions of Arctic and alpine ecosystems, we included new parameterizations for peat soils and switches to start and stop photosynthesis to simulate dormancy during winter. Cold temperatures, freezing soils, the hibernation behavior of vegetation to not perform photosynthesis in the winter, and the varying behavior of soils with permafrost conditions required updates to model formulation in order to apply MLCan to this new region. The updates to MLCan are validated for all sites in Figures S1 – S3.

Soil. Soil properties, such as sand, clay, and organic material content and hydraulic and thermal conductivity are parameterized and held constant throughout the model simulations. Due to the presence of peat soils in the Arctic, we implemented formulations for the thermal conductivity of peat soils instead of basing them entirely off of sand/clay percentages. The thermal conductivity model was based on equations from Zhao et al[42], and parameterizations for thermal and hydraulic conductivity were based on Wu et al[43] and Krogh et al[27]. To further take into account the behavior of Arctic permafrost, the model was altered to turn off plant-soil uptake when a given soil layer was frozen, specifically when the soil temperature within the layer of the subsurface was determined to be below -1°C.

Canopy. We created dynamic switches to stop photosynthetic activity during winter when the mean air temperature in the canopy over the previous 24 hours drops below a certain threshold (-7°C) and restart when it returns above a certain threshold (3°C)[44]. Based on site-specific literature, different photosynthesis stop (-3°C) and start (5°C) thresholds were used for NR1 vegetation[11]. Additional constraints preventing photosynthesis from occurring when the top layer of the soil is frozen or the snow depth is greater than the canopy height are also included[45]. We do not close the stomata or change the respiration routine since literature indicates that respiration can occur during winter, even when photosynthesis is not occurring[46]. The periods of time when photosynthesis was active were validated based on site-specific literature when available.

Further, due to the extended periods of snowpack in the regions studied, new parameters were created to demonstrate the change in canopy reflectance with snow[47]. These *new snow reflection coefficients* vary based on the surface (i.e., canopy, peat soil, sandy soil) and the type of radiation (i.e., PAR, NIR) and are summarized in Table S1.

Ecosystem-wide. Due to the sensitivity of latent heat of vaporization (Lv) in colder regions, we implemented dynamic Lv based on air temperature[48] rather than keeping it as a static parameter. We implemented a bi-directional formulation for estimating temperature and vapor pressure values from observed fluxtower measurements. Since counterfactuals were constructed at the alpine/Arctic sites, fluxtower measurements above alpine/Arctic shrubs were located below the height of the simulated trees and, consequently, the ecosystem height (see Table S1). This ecosystem height was used as the upper bound of the control volume for each site pair such that shorter canopy and flux tower heights (i.e., alpine/Arctic tundra or meadow) could be compared directly with taller ecosystems (i.e., subalpine/subArctic forest). Since fluxtower measurements were collected below the ecosystem height for the alpine/Arctic sites and above the ecosystem height for the subalpine/sub-Arctic sites, the new formulation calculates the estimated temperature and vapor pressure deficit at the ecosystem height from the observed values from either above or below using similarity theory.

Supplementary Figures

The main text presents several figures with data or results from only one or two of the locations studied. This includes the average daily work by scenario throughout the year; the daily snow depth, average daily photosynthetic rate, and average daily respiration rate for each scenario throughout the year; and 3-D plots of temperature gradient, leaf area index, and work for all scenarios. The data for the remaining sites are presented in Figs. S5 – S7, respectively.

Table S1: Model Parameters

**Location**	**Italian Alps**			**Western Canadian Taiga-Tundra**			**United States Rocky Mountains**		
**Site**	**Lav**		**MBo**	**HPC**		**TVC**	**NR1**		**T-Van**
Functional Group	UN	OT	UN	UN	OT	UN	UN	OT	UN
Site Information									
Latitude	45.9562°N[a]		46.0147°N[a]	68.3203°N[b]		68.7462°N[b]	40.0329°N[c]		40.053°N[d]
Longitude	11.2813°E[a]		11.0458°E[a]	133.519°W[b]		133.502°W[b]	105.546°W[c]		105.586°W[d]
Elevation (m)	1349[a]		1553[a]	80[b]		85[b]	3050[c]		3503[d]
Fluxtower Height (m)	36[e]		2.5[f]	15		4[g]	21.5[c]		3[d]
Canopy Height (m)	4[e]	36[e]	0.3[a]	1.4[h]	8[h]	0.3[h]	0.15[j]	12[j]	0.05[d]
Ecosystem Height[k] (m)	36		36	8		8	12		12
Percent Sand	29.5[e]		37[l]	35[m]		35[m]	72[n]		55.9[o]
Percent Clay	26.5[e]		21[l]	40[m]		40[m]	1[n]		16.3[o]
Respiration $Q10$ (mol/m^{2}s)	2.9[p,q]		4[p]	3.2[p]		9[p]	2.6[p,r]		3.1[s]
Surface Roughness Length (m)	10.8[t]		0.125[u]	1.1[v]		0.08[w]	1.79[j]		0.1[x]
Ambient CO_2[y] (ppm)	393		393	405		405	405[z]		405[z]
Leaf Properties									
Vc_{max} (μmol/m^{2}s)	42[aa]	45[bb]	37.93[cc]	78.1[dd]	44.6[ee]	78.1[aa]	43[ff]	11[gg]	67.81[hh]
J_{max} (μmol/m^{2}s)	78[aa]	100[bb]	84[q]	119.4[dd]	43.7[ee]	119.4[aa]	91[ff]	51.7[cc]	149.86[ii]
Rd_{25} (μmol/m^{2}s)	1[jj]	2.142[bb]	1.4[q]	1.47[kk]	2.32[ll]	2.94[kk]	0.6[mm]	0.9[mm]	1.4[q]
Ball-Berry Slope[nn]	15.6	5.7	15.6	15.6	5.7	15.6	15.6	5.7	9[z]
Ball-Berry Intercept[nn] (mol/m^{2}s)	0.02	0.02	0.02	0.02	0.02	0.02	0.02	0.02	0.002[z]
Reflectance									
PAR reflection coefficient[oo]	0.05		0.1	0.05		0.05	0.05		0.05
NIR reflection coefficient[oo]	0.2		0.35	0.4		0.2	0.2		0.3
Soil reflection coefficient[oo,pp]	0.15		0.3	0.17[qq]		0.3	0.17		0.2
New Snow PAR reflection coefficient[oo]	0.1		0.15	0.1		0.2	0.07		0.1
New Snow NIR reflection coefficient[oo]	0.1		0.3	0.4		0.25	0.1		0.2
New Snow Soil reflection coefficient[oo,rr]	0.6		0.8	0.6		0.8	0.3		0.6
Root Properties									
Root Depth (m)	0.3[tt]	0.5[tt]	0.3[tt]	0.5[i,uu]	0.25[i,vv]	0.5[i,uu]	0.3	0.5[ww]	0.4
z_{50} [ss]	0.03[tt]	0.15[tt]	0.03[tt]	0.1[i,uu]	0.1[i,vv]	0.1[i,uu]	0.03	0.15[ww]	0.03
z_{95} [ss]	0.2[tt]	0.3[tt]	0.2[tt]	0.4[i,uu]	0.19[i,vv]	0.4[i,uu]	0.2	0.3[ww]	0.2

UN refers to the understory, and OT refers to the overstory trees.

[a] Tudoroiu et al, 2016[49]
[b] AmeriFlux Network: CA-HPC & CA-TVC.
[c] Bowling et al, 2018[11]
[d] Knowles et al, 2012[7]
[e] Marcolla et al, 2003[22]
[f] Ramírez-Cuesta et al, 2018[50]
[g] Helbig et al, 2016[51]
[h] Krogh et al, 2017[27]
[j] Turnipseed et al, 2002[13]
[k] Taken as maximum canopy height for each region site pair
[l] Groenendij, 2012[52]
[m] Marsh et al, 2010[53]. Arctic peat was modeled differently; see *Soil* section in *MLCan Model Updates*.
[n] Burns et al, 2018[54]
[o] Seastedt & Adams, 2001[55]
[p] Chen & Tian, 2005[56]
[q] Urban et al, 2007[57]
[r] Sacks et al, 2007[58]
[s] Tjoelker et al, 2001[59]
[t] Cescatti & Marcolla, 2004[60]
[u] Marcolla & Cescatti, 2005[61]
[v] Kelliher et al, 1993[62]
[w] Beringer et al, 2005[63]
[x] Litaor et al, 2008[64]
[y] All ecosystems (aside from the US sites) use the median global average ambient CO_2 of all years modeled for each region[65]
[z] Wentz et al, 2019[66]
[aa] Liozon et al, 2000[67]
[bb] Robakowski et al, 2005[68]
[cc] Wohlfahrt et al, 1999[69]
[dd] Bubier et al, 2011[70]
[ee] Way & Sage, 2008[71]
[ff] Wullschleger, 1993[72]
[gg] Tomaszewski & Sievering, 2007[73]
[hh] Cannone et al, 2016[74]
[ii] Inferred from Fan et al, 2011[75]
[jj] Keiner et al, 2015[76]
[kk] Calculated from Smith & Hadley, 1974[77] & Marsh et al, 2010[53]
[ll] Goulden et al, 1997[78]
[mm] Reich et al, 1998[79]
[nn] Ueyama et al, 2018[80]
[oo] Inferred from Heinilä et al, 2019[47]
[pp] Stoner et al, 1980[81]
[qq] Hashimoto et al, 2021[82]
[rr] Wiscomb & Warren, 1980[81]
[ss] Schenk & Jackson[83]
[tt] Wohlfahrt et al, 1998[84], Martinez et al[85], & Tudoroiu et al, 2016[49]
[uu] Hébert & Thiffault, 2011[86]
[vv] Lieffers & Rothwell, 1986[87]; Flanagan & Van Cleve, 1977[88]
[ww] Burns et al, 2015[12]

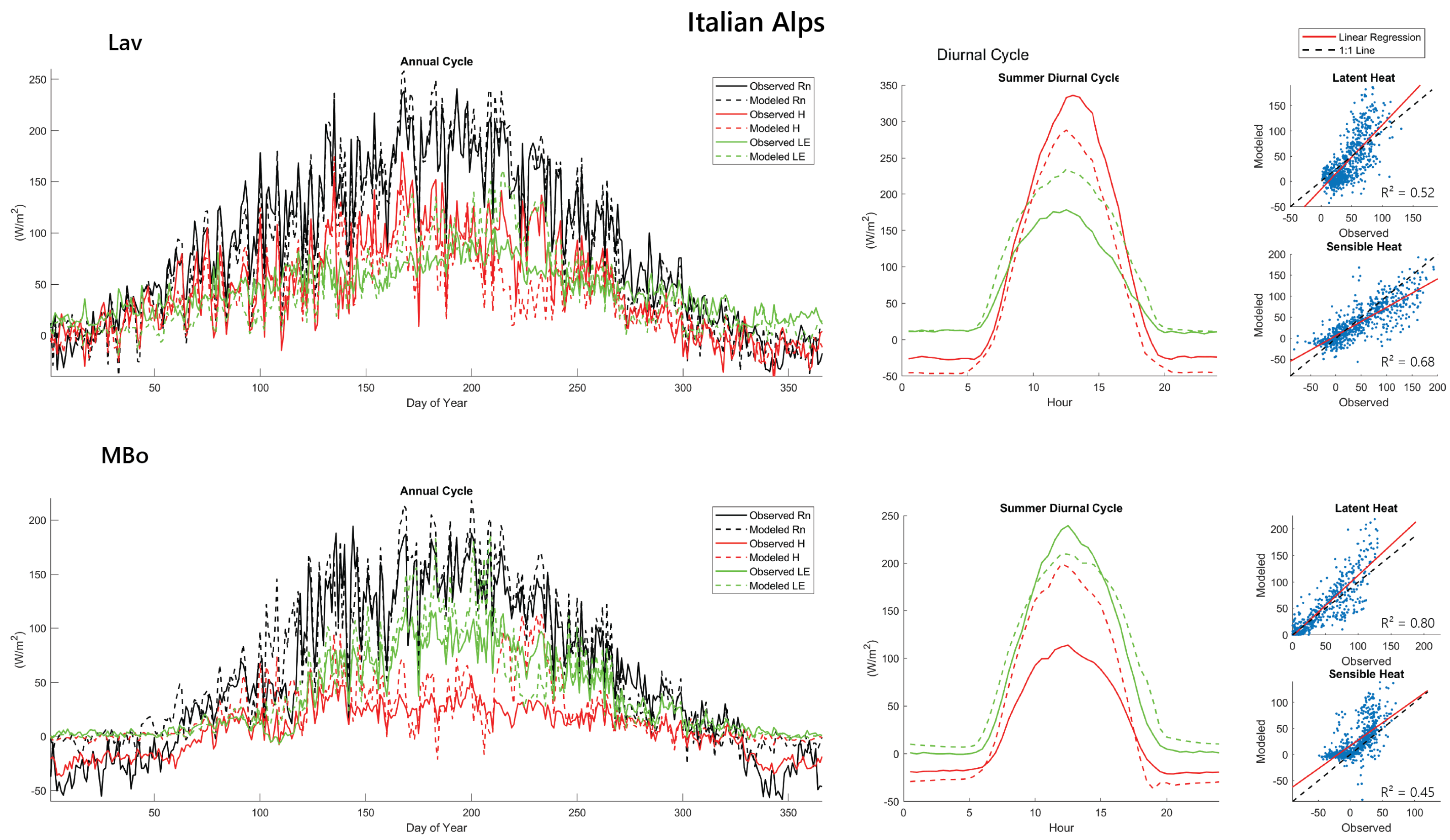

Figure S1: Model validation for the Italian Alps sites: Lav (subalpine forest) and MBo (alpine meadow). Graphs include average annual cycle, average diurnal cycle during summer, and the 1:1 plot of all shared days within the study period, 2012-2013.

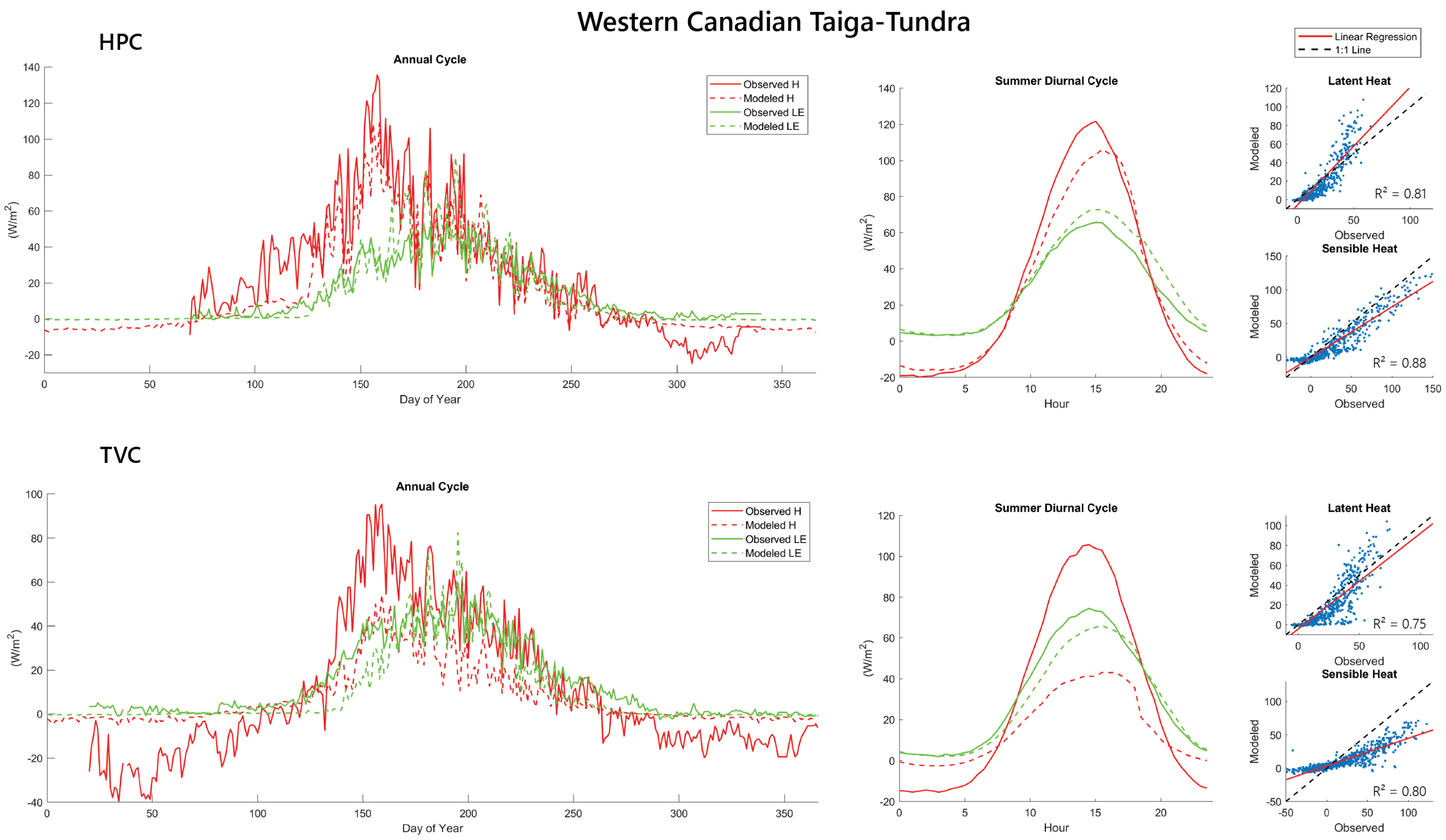

Figure S2: Model validation for the Western Canadian Taiga-Tundra sites: HPC (subArctic forest) and TVC (Arctic tundra). Graphs include average annual cycle, average diurnal cycle during summer, and the 1:1 plot of all shared days within the study period, 2016-2018.

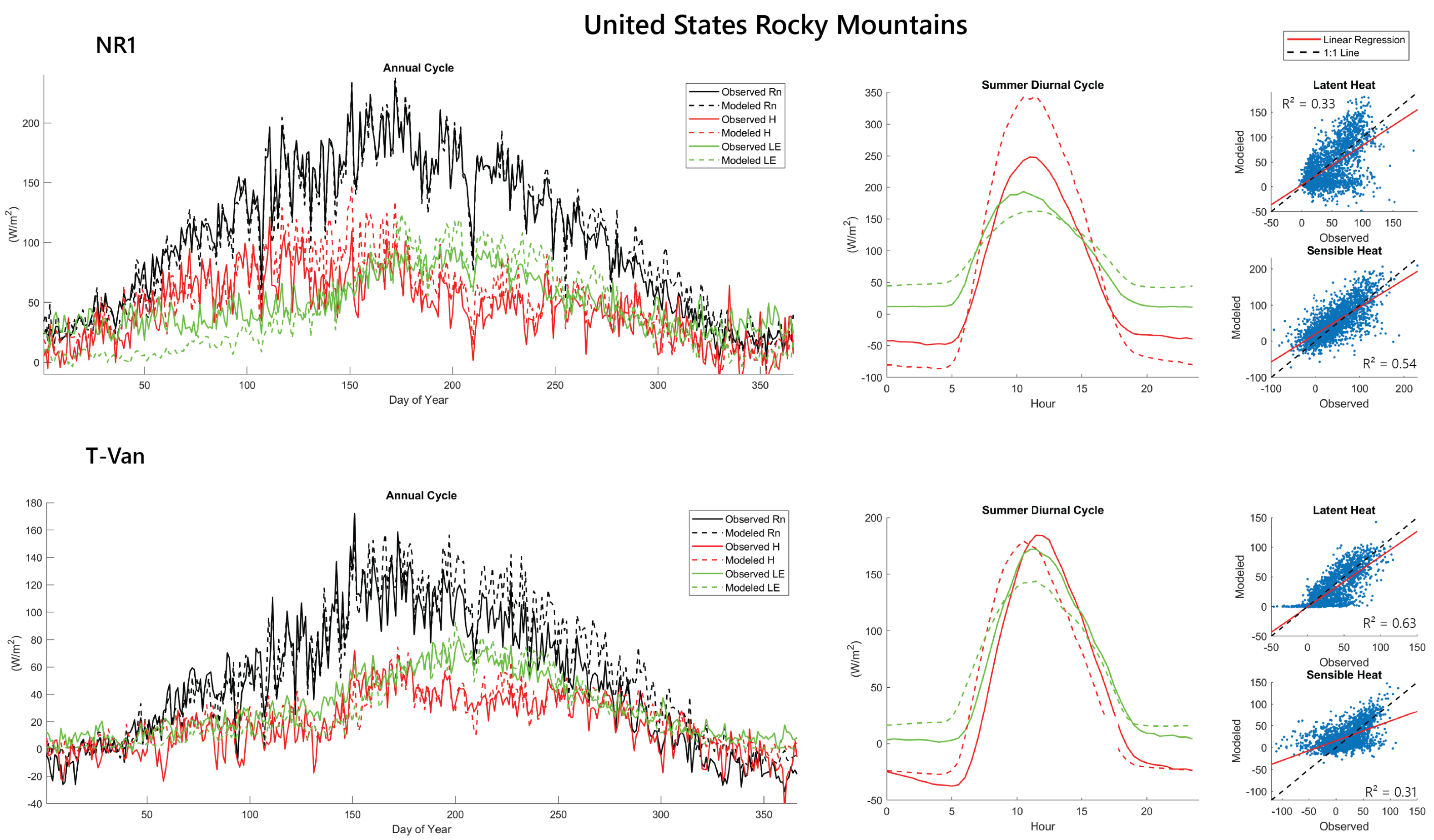

Figure S3: Model validation for the two United States Rocky Mountains sites: NR1 (subalpine forest) and TVan (alpine fellfield). Graphs include average annual cycle, average diurnal cycle during summer, and the 1:1 plot of all shared days within the study period, 2008-2013.

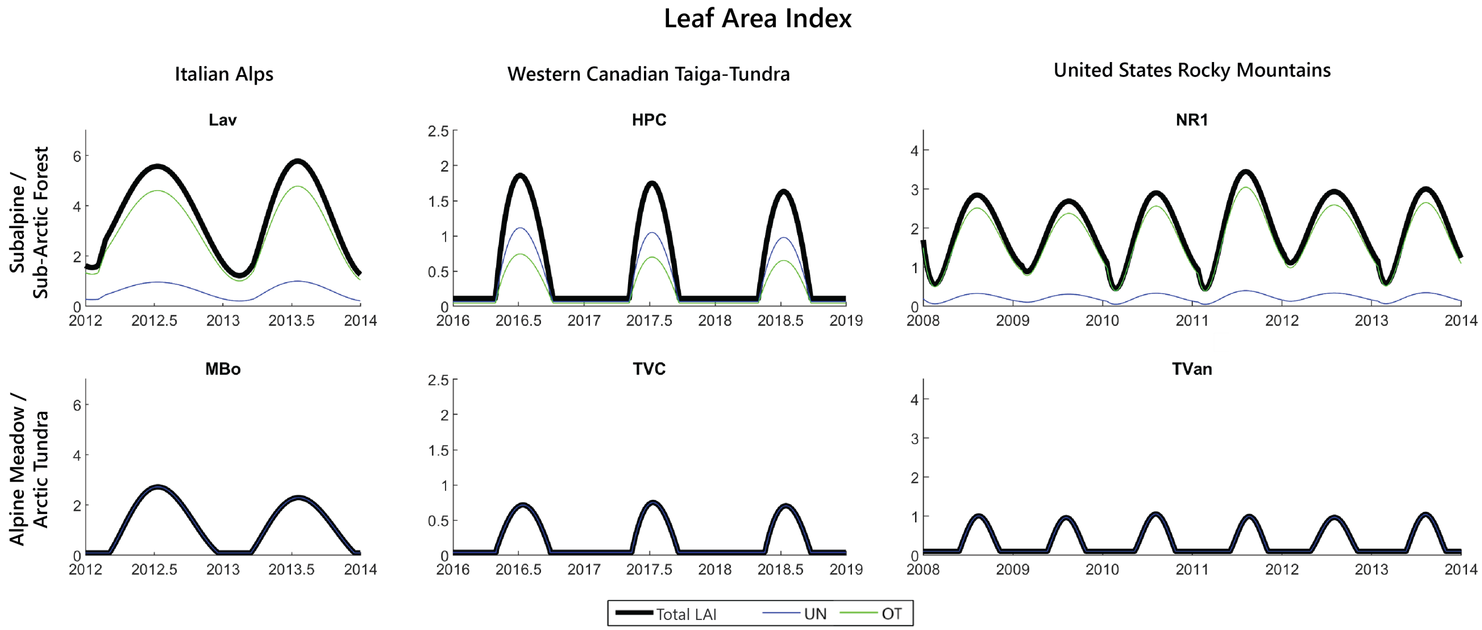

Figure S4: Leaf area index (LAI) for all sites interpolated from MODIS[14–20] and calibrated and partitioned based on site documentation. UN refers to the understory, and OT refers to the overstory trees.

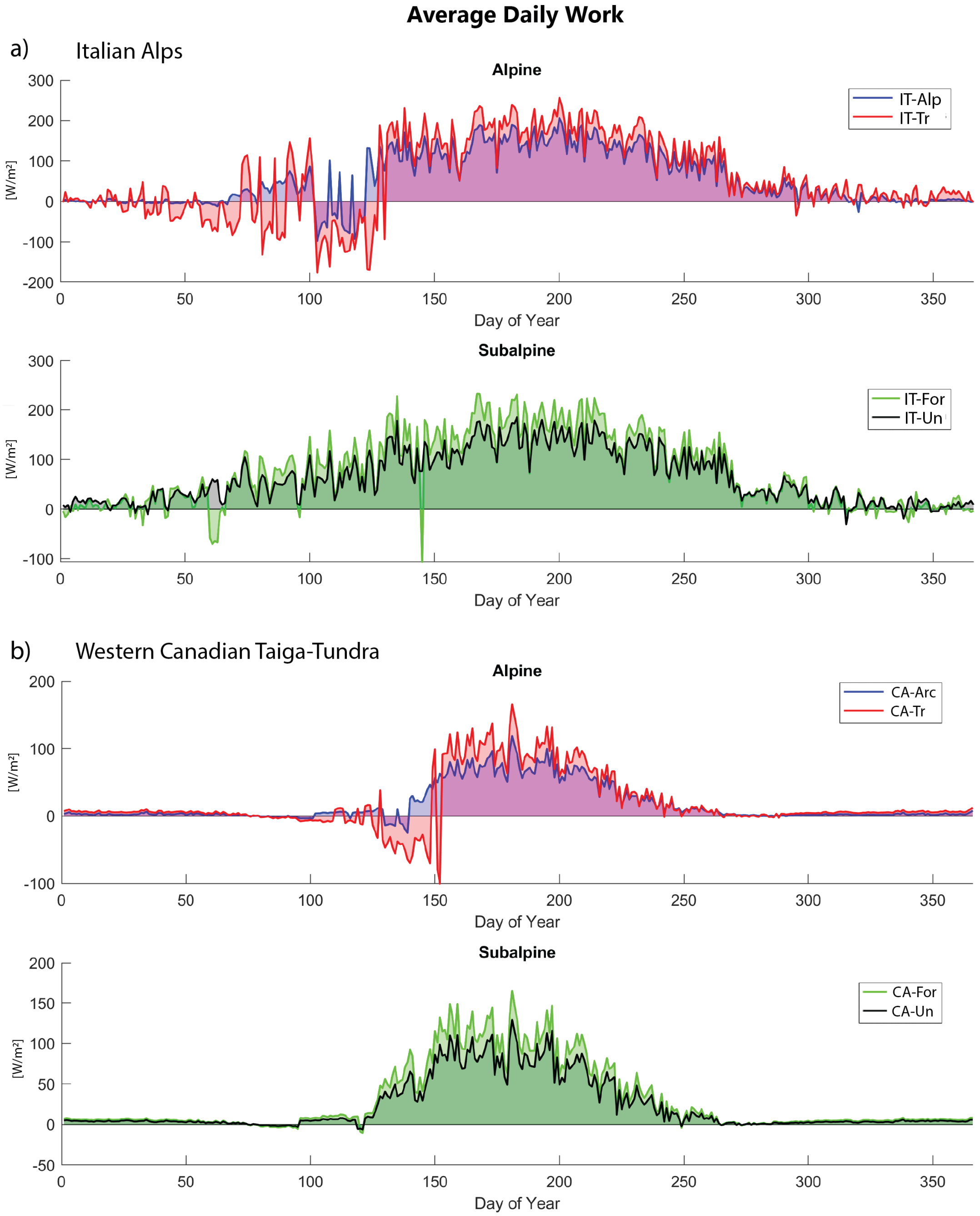

Figure S5: Annual time series of work, averaged daily, for the entire study period for scenarios in the (a) Italian Alps (2012-2013) and (b) Western Canadian Taiga-Tundra (2016-2018). Refer to Figure 4 of the main text for information on the United States Rocky Mountains scenarios.

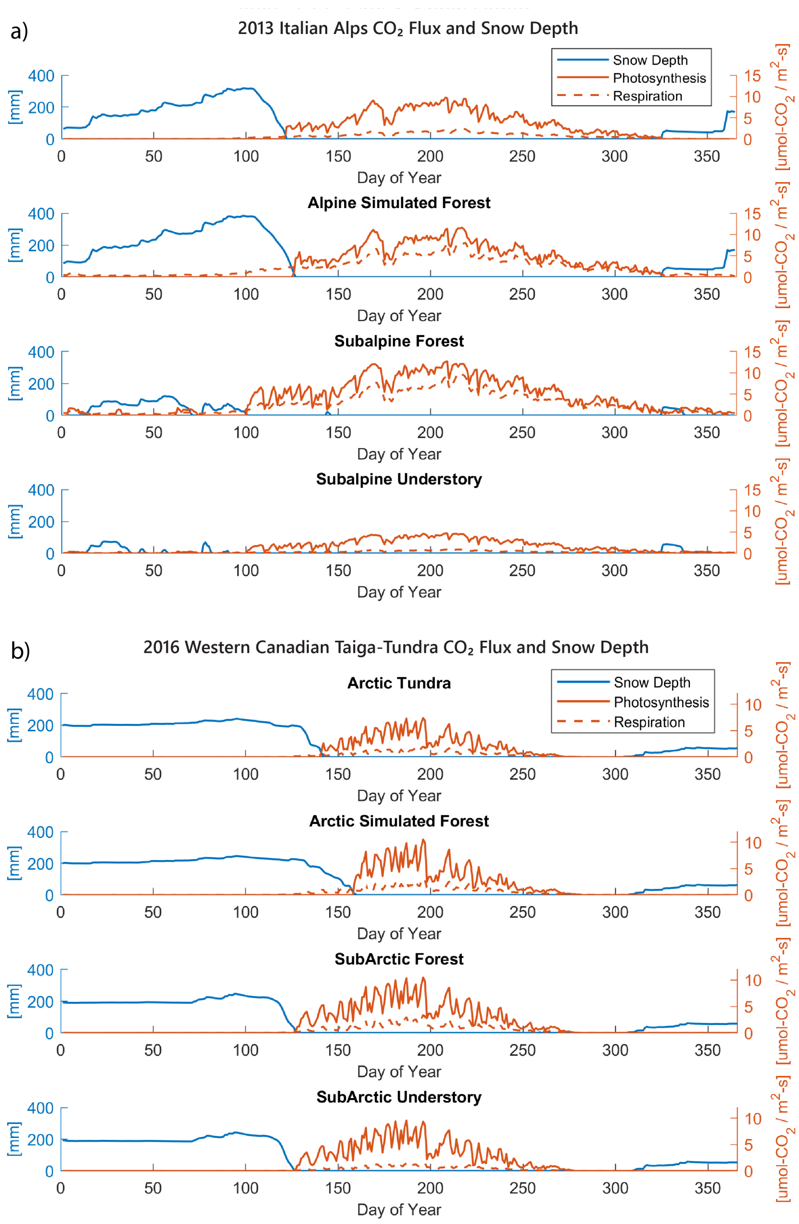

Figure S6: Average daily leaf CO_2 flux and snow depth for representative years at the Italian Alps and Western Canadian Taiga-Tundra. Refer to Figure 5 of the main text for information on the United States Rocky Mountains scenarios. (a) 2013 daily timeseries of snow depth (*blue*) and leaf CO_2 flux – the averaged daily photosynthetic CO_2 uptake (*orange solid line*) and above-ground autotrophic respiration (*orange dotted line*) – for the Italian Alps scenarios. (b) 2013 daily timeseries of snow depth (*blue*) and leaf CO_2 flux – the averaged daily photosynthetic CO_2 uptake (*orange solid line*) and above-ground autotrophic respiration (*orange dotted line*) – for the Italian Alps scenarios.

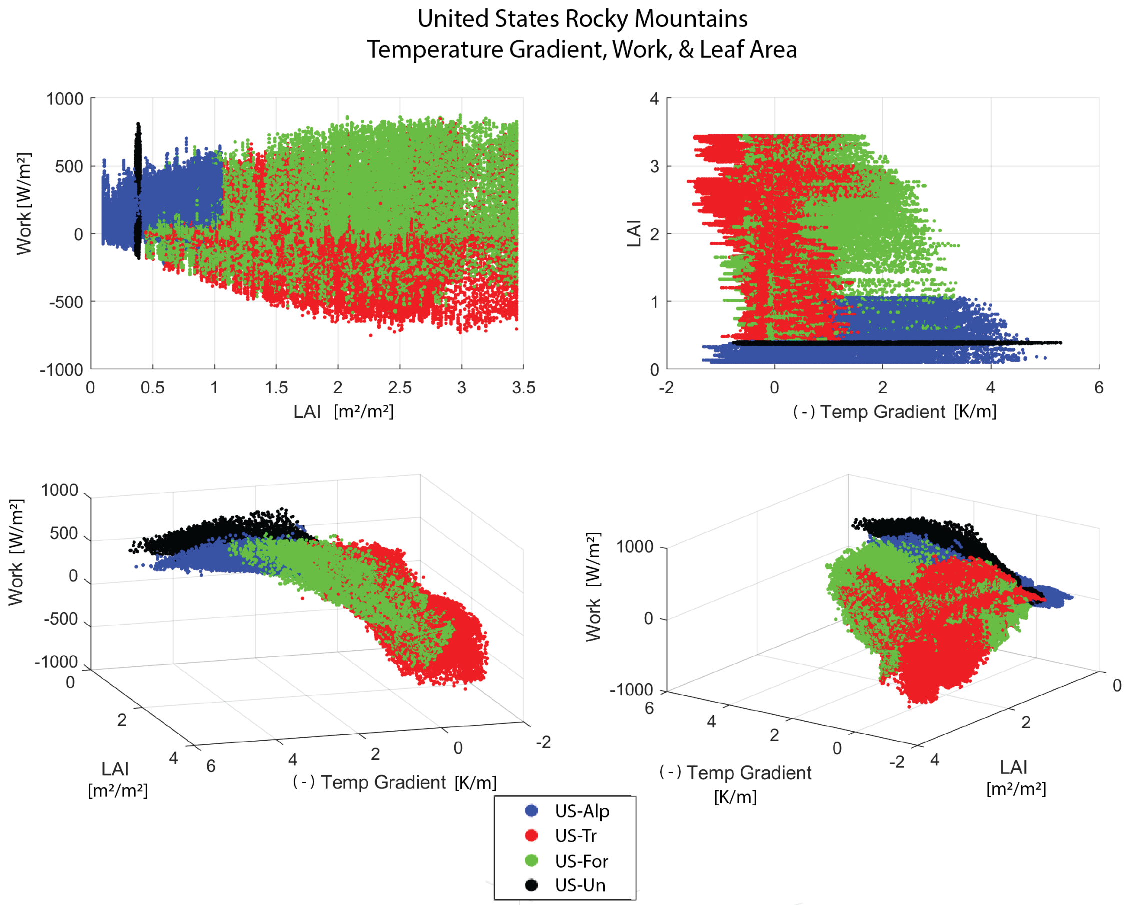

Figure S7: Four projected views of the work, temperature gradient, leaf area index (LAI) 3D plot for the (a) Italian Alps and (b) United States Rocky Mountains scenarios. Refer to Figure 6 of the main text for information on the Western Canadian Taiga-Tundra scenarios. The negative of the resultant temperature gradient is plotted. Thus, positive values refer to negative temperature gradients such that larger values indicate stronger declines in temperature from the earth surface to the atmosphere. Negative values indicate positive temperature gradients, or temperature inversions. The 3D views show the transition from flatter curves to greater marginal increases in work with increases in temperature gradient as more LAI is modeled for each set of environmental conditions (i.e., alpine, subalpine). The simulated alpine forest scenario exhibits considerable negative work values since the LAI is beyond the supported limit of the local environmental conditions.

References

1. Gianelle, D., Zampedri, R., Cavagna, M. & Sottocornola, M. FLUXNET2015 IT-Lav Lavarone, Dataset. DOI: 10.18140/FLX/1440169 (2003-2014).
2. Gianelle, D., Cavagna, M., Zampedri, R. & Marcolla, B. FLUXNET2015 IT-MBo Monte Bondone. DOI: 10.18140/FLX/1440170 (2016).
3. Wutzler, T. *et al.* Basic and extensible post-processing of eddy covariance flux data with reddyproc. *Biogeosciences Discuss.* **15**, 5015–5030, DOI: 10.5194/bg-15-5015-2018 (2018).
4. Sonnentag, O. & Marsh, P. AmeriFlux CA-HPC Havikpak Creek, Ver. 1-5, AmeriFlux AMP, (Dataset). DOI: 10.17190/AMF/1773392 (2021).
5. Sonnentag, O. & Marsh, P. AmeriFlux CA-TVC Trail Valley Creek, Ver. 1-5, AmeriFlux AMP, (Dataset). DOI: 10.17190/AMF/1767831 (2021).
6. Ritschel, C., Ulbrich, U., Névir, P. & Rust, H. W. Precipitation extremes on multiple timescales–bartlett–lewis rectangular pulse model and intensity–duration–frequency curves. *Hydrol. Earth Syst. Sci.* **21**, 6501, DOI: 10.5194/hess-21-6501-2017 (2017).
7. Knowles, J. F., Blanken, P. D., Williams, M. W. & Chowanski, K. M. Energy and surface moisture seasonally limit evaporation and sublimation from snow-free alpine tundra. *Agric. For. Meteorol.* **157**, 106–115, DOI: 10.1016/j.agrformet.2012.01.017 (2012).
8. Blanken, P. D., Monson, R. K., Burns, S. P., Bowling, D. R. & Turnipseed, A. A. AmeriFlux US-NR1 Niwot Ridge Forest (LTER NWT1), Dataset. DOI: 10.17190/AMF/1246088 (1998-).
9. Knowles, J. Infilled climate and heat flux data for Tvan towers data loggers (CR3000), 2008 - ongoing. ver 1. Environmental Data Initiative. DOI: 10.6073/pasta/10fb65e51cd04631bb80c82288b5c51a (2018).
10. Jennings, K., Kittel, T. & Molotch, N. Infilled climate data for C1, Saddle, and D1, 1990 - 2013, hourly. ver 1. Environmental Data Initiative. DOI: 10.6073/pasta/1538ccf520d89c7a11c2c489d973b232 (2019).
11. Bowling, D. R. *et al.* Limitations to winter and spring photosynthesis of a rocky mountain subalpine forest. *Agric. For. Meteorol.* **252**, 241–255, DOI: 10.1016/j.agrformet.2018.01.025 (2018).
12. Burns, S., Blanken, P., Turnipseed, A., Hu, J. & Monson, R. The influence of warm-season precipitation on the diel cycle of the surface energy balance and carbon dioxide at a colorado subalpine forest site. *Biogeosciences* **12**, 7349–7377, DOI: 10.5194/bg-12-7349-2015 (2015).
13. Turnipseed, A., Blanken, P., Anderson, D. & Monson, R. K. Energy budget above a high-elevation subalpine forest in complex topography. *Agric. For. Meteorol.* **110**, 177–201, DOI: 10.1016/S0168-1923(01)00290-8 (2002).
14. Myneni, R., Knyazikhin, Y. & Park, T. MCD15A2H MODIS/Terra+Aqua Leaf Area Index/FPAR 8-Day L4 Global 500m SIN Grid V006. *NASA EOSDIS Land Process. DAAC* DOI: 10.5067/MODIS/MCD15A2H.006 (2015).
15. ORNL DAAC. Fixed sites subsetting and visualization tool, DOI: 10.3334/ORNLDAAC/1567 (2018). ORNL DAAC, Oak Ridge, Tennessee, USA. Accessed August 03, 2020. Subset obtained for MCD15A2H product at site id ‘it_trentino_altoadige_lavarone’.
16. ORNL DAAC. Fixed sites subsetting and visualization tool, DOI: 10.3334/ORNLDAAC/1567 (2018). ORNL DAAC, Oak Ridge, Tennessee, USA. Accessed August 03, 2020. Subset obtained for MCD15A2H product at site id ‘it_trentino_altoadige_monte_bondone’.
17. ORNL DAAC. Fixed sites subsetting and visualization tool, DOI: 10.3334/ORNLDAAC/1567 (2018). ORNL DAAC, Oak Ridge, Tennessee, USA. Accessed August 03, 2020. Subset obtained for MCD15A2H product at site id ‘ca_northwestterritories_havikpak_creek’.

18. ORNL DAAC. Fixed sites subsetting and visualization tool, DOI: 10.3334/ORNLDAAC/1567 (2018). ORNL DAAC, Oak Ridge, Tennessee, USA. Accessed August 03, 2020. Subset obtained for MCD15A2H product at site id 'us_alaska_trail_valley_creek'.

19. ORNL DAAC. Fixed sites subsetting and visualization tool, DOI: 10.3334/ORNLDAAC/1567 (2018). ORNL DAAC, Oak Ridge, Tennessee, USA. Accessed August 03, 2020. Subset obtained for MCD15A2H product at site id 'us_colorado_niwot_ridge'.

20. ORNL DAAC. Fixed sites subsetting and visualization tool, DOI: 10.3334/ORNLDAAC/1567 (2018). ORNL DAAC, Oak Ridge, Tennessee, USA. Accessed August 03, 2020. Subset obtained for MCD15A2H product at site id 'us_colorado_niwot_ridge_mountain_research_station'.

21. Richardson, M. & Kumar, P. Discerning the thermodynamic feasibility of the spontaneous coexistence of multiple functional vegetation groups. *Nat. Sci. Reports* (2020).

22. Marcolla, B., Pitacco, A. & Cescatti, A. Canopy architecture and turbulence structure in a coniferous forest. *Boundary-layer meteorology* **108**, 39–59 (2003).

23. Lemoine, D., Cochard, H. & Granier, A. Within crown variation in hydraulic architecture in beech (fagus sylvatica l): evidence for a stomatal control of xylem embolism. *Annals forest science* **59**, 19–27 (2002).

24. Gianelle, D., Vescovo, L., Marcolla, B., Manca, G. & Cescatti, A. Ecosystem carbon fluxes and canopy spectral reflectance of a mountain meadow. *Int. J. Remote. Sens.* **30**, 435–449 (2009).

25. Knowles, J. F., Burns, S. P., Blanken, P. D. & Monson, R. K. Fluxes of energy, water, and carbon dioxide from mountain ecosystems at niwot ridge, colorado. *Plant Ecol. & Divers.* **8**, 663–676, DOI: 10.1080/17550874.2014.904950 (2015).

26. Blanken, P. D. *et al.* A comparison of water and carbon dioxide exchange at a windy alpine tundra and subalpine forest site near niwot ridge, colorado. *Biogeochemistry* **95**, 61–76, DOI: 10.1007/s10533-009-9325-9 (2009).

27. Krogh, S. A., Pomeroy, J. W. & Marsh, P. Diagnosis of the hydrology of a small arctic basin at the tundra-taiga transition using a physically based hydrological model. *J. Hydrol.* **550**, 685–703, DOI: 10.1016/j.jhydrol.2017.05.042 (2017).

28. Ryu, Y. *et al.* On the correct estimation of effective leaf area index: Does it reveal information on clumping effects? *Agric. For. Meteorol.* **150**, 463–472, DOI: 10.1016/j.agrformet.2010.01.009 (2010).

29. Sonnentag, O. *et al.* Mapping tree and shrub leaf area indices in an ombrotrophic peatland through multiple endmember spectral unmixing. *Remote. Sens. Environ.* **109**, 342–360, DOI: 0.1016/j.rse.2007.01.010 (2007).

30. Sonnentag, O., Talbot, J., Chen, J. & Roulet, N. Using direct and indirect measurements of leaf area index to characterize the shrub canopy in an ombrotrophic peatland. *Agric. For. Meteorol.* **144**, 200–212, DOI: 10.1016/j.agrformet.2007.03.001 (2007).

31. Cava, D. & Katul, G. G. Spectral short-circuiting and wake production within the canopy trunk space of an alpine hardwood forest. *Boundary-layer meteorology* **126**, 415–431 (2008).

32. Alexander, M. E. & (Canada), N. F. C. *Characterizing the jack pine-black spruce fuel complex of the International Crown Fire Modelling Experiment (ICFME)* (Canadian Forest Service, Northern Forestry Centre, 2004).

33. Weibull, W. Wide applicability. *J. applied mechanics* **103**, 293–297 (1951).

34. Bartkute, V. & Sakalauskas, L. The method of three-parameter weibull distribution estimation. *Acta et commentationes Univ. Tartuensis de mathematica* **12**, 65–78 (2008).

35. Drewry, D. *et al.* Ecohydrological responses of dense canopies to environmental variability: 1. interplay between vertical structure and photosynthetic pathway. *J. Geophys. Res. Biogeosciences* **115**, DOI: 10.1029/2010JG001340 (2010).

36. Le, P. V., Kumar, P. & Drewry, D. T. Implications for the hydrologic cycle under climate change due to the expansion of bioenergy crops in the midwestern united states. *Proc. Natl. Acad. Sci.* DOI: 10.1073/pnas.1107177108 (2011).

37. Le, P. V., Kumar, P., Drewry, D. T. & Quijano, J. C. A graphical user interface for numerical modeling of acclimation responses of vegetation to climate change. *Comput. & Geosci.* **49**, 91–101, DOI: 10.1016/j.cageo.2012.07.007 (2012).

38. Quijano, J. C., Kumar, P., Drewry, D. T., Goldstein, A. & Misson, L. Competitive and mutualistic dependencies in multispecies vegetation dynamics enabled by hydraulic redistribution. *Water Resour. Res.* **48**, DOI: 10.1029/2011WR011416 (2012).

39. Quijano, J. C., Kumar, P. & Drewry, D. T. Passive regulation of soil biogeochemical cycling by root water transport. *Water Resour. Res.* **49**, 3729–3746, DOI: 10.1002/wrcr.20310 (2013).

40. Quijano, J. C. & Kumar, P. Numerical simulations of hydraulic redistribution across climates: The role of the root hydraulic conductivities. *Water Resour. Res.* **51**, 8529–8550, DOI: 10.1002/2014WR016509 (2015).

41. Lee, E. *et al.* Impact of hydraulic redistribution on multispecies vegetation water use in a semiarid savanna ecosystem: An experimental and modeling synthesis. *Water Resour. Res.* **54**, 4009–4027, DOI: 10.1029/2017WR021006 (2018).

42. Zhao, Y. *et al.* A new thermal conductivity model for sandy and peat soils. *Agric. For. Meteorol.* **274**, 95–105, DOI: 10.1016/j.agrformet.2019.04.004 (2019).

43. Wu, Y., Verseghy, D. L. & Melton, J. R. Integrating peatlands into the coupled Canadian Land Surface Scheme (CLASS) v3. 6 and the Canadian Terrestrial Ecosystem Model (CTEM) v2.0. *Geosci. Model. Dev.* **9**, DOI: 10.5194/gmd-9-2639-2016 (2016).

44. Sevanto, S. *et al.* Wintertime photosynthesis and water uptake in a boreal forest. *Tree Physiol.* **26**, 749–757, DOI: 10.1093/treephys/26.6.749 (2006).

45. Bergh, J., McMurtrie, R. E. & Linder, S. Climatic factors controlling the productivity of norway spruce: a model-based analysis. *For. ecology management* **110**, 127–139, DOI: 10.1016/S0378-1127(98)00280-1 (1998).

46. Grogan, P. Cold season respiration across a low arctic landscape: the influence of vegetation type, snow depth, and interannual climatic variation. *Arctic, antarctic, alpine research* **44**, 446–456, DOI: 10.1657/1938-4246-44.4.446 (2012).

47. Heinilä, K. *et al.* Reflectance variation in boreal landscape during the snow melting period using airborne imaging spectroscopy. *Int. J. Appl. Earth Obs. Geoinformation* **76**, 66–76, DOI: 10.1016/j.jag.2018.10.017 (2019).

48. Henderson-Sellers, B. A new formula for latent heat of vaporization of water as a function of temperature. *Q. J. Royal Meteorol. Soc.* **110**, 1186–1190, DOI: 10.1002/qj.49711046626 (1984).

49. Tudoroiu, M. *et al.* Negative elevation-dependent warming trend in the eastern alps. *Environ. Res. Lett.* **11**, 044021 (2016).

50. Ramírez-Cuesta, J. M., Vanella, D., Consoli, S., Motisi, A. & Minacapilli, M. A satellite stand-alone procedure for deriving net radiation by using SEVIRI and MODIS products. *Int. journal applied earth observation geoinformation* **73**, 786–799 (2018).

51. Helbig, M. *et al.* Addressing a systematic bias in carbon dioxide flux measurements with the ec150 and the irgason open-path gas analyzers. *Agric. For. Meteorol.* **228**, 349–359, DOI: 10.1016/j.agrformet.2016.07.018 (2016).

52. Groenendijk, M. *Boxing Nature: Global generalities in terrestrial ecosystem photosynthesis and transpiration*. Ph.D. thesis, Vrije Universiteit Amsterdam (2012). Naam instelling promotie: VU Vrije Universiteit Naam instelling onderzoek: VU Vrije Universiteit.

53. Marsh, P., Bartlett, P., MacKay, M., Pohl, S. & Lantz, T. Snowmelt energetics at a shrub tundra site in the western canadian arctic. *Hydrol. Process.* **24**, 3603–3620, DOI: 10.1002/hyp.7786 (2010).

54. Burns, S. P. *et al.* A comparison of the diel cycle of modeled and measured latent heat flux during the warm season in a colorado subalpine forest. *J. Adv. Model. Earth Syst.* **10**, 617–651, DOI: 10.1002/2017MS001248 (2018).

55. Seastedt, T. & Adams, G. A. Effects of mobile tree islands on alpine tundra soils. *Ecology* **82**, 8–17, DOI: doi.org/10.1890/0012-9658(2001)082[0008:EOMTIO]2.0.CO;2 (2001).

56. Chen, H. & Tian, H.-Q. Does a general temperature-dependent Q10 model of soil respiration exist at biome and global scale? *J. integrative plant biology* **47**, 1288–1302, DOI: 10.1111/j.1744-7909.2005.00211.x (2005).

57. Urban, O. *et al.* Temperature dependences of carbon assimilation processes in four dominant species from mountain grassland ecosystem. *Photosynthetica* **45**, 392–399 (2007).

58. Sacks, W. J., Schimel, D. S. & Monson, R. K. Coupling between carbon cycling and climate in a high-elevation, subalpine forest: a model-data fusion analysis. *Oecologia* **151**, 54–68, DOI: 10.1007/s00442-006-0565-2 (2007).

59. Tjoelker, M. G., Oleksyn, J. & Reich, P. B. Modelling respiration of vegetation: evidence for a general temperature-dependent Q10. *Glob. Chang. Biol.* **7**, 223–230, DOI: 10.1046/j.1365-2486.2001.00397.x (2001).

60. Cescatti, A. & Marcolla, B. Drag coefficient and turbulence intensity in conifer canopies. *Agric. forest meteorology* **121**, 197–206 (2004).

61. Marcolla, B. & Cescatti, A. Experimental analysis of flux footprint for varying stability conditions in an alpine meadow. *Agric. For. Meteorol.* **135**, 291–301 (2005).

62. Kelliher, F., Leuning, R. & Schulze, E. D. Evaporation and canopy characteristics of coniferous forests and grasslands. *Oecologia* **95**, 153–163, DOI: 10.1007/BF00323485 (1993).

63. Beringer, J., Chapin III, F. S., Thompson, C. C. & McGuire, A. D. Surface energy exchanges along a tundra-forest transition and feedbacks to climate. *Agric. For. Meteorol.* **131**, 143–161, DOI: 10.1016/j.agrformet.2005.05.006 (2005).

64. Litaor, M., Williams, M. & Seastedt, T. Topographic controls on snow distribution, soil moisture, and species diversity of herbaceous alpine vegetation, niwot ridge, colorado. *J. Geophys. Res. Biogeosciences* **113**, DOI: 10.1029/2007JG000419 (2008).

65. Dlugokencky, E. & Tans, P. NOAA/GML (www.esrl.noaa.gov/gmd/ccgg/trends/). (2020).

66. Wentz, K. F., Neff, J. C. & Suding, K. N. Leaf temperatures mediate alpine plant communities' response to a simulated extended summer. *Ecol. evolution* **9**, 1227–1243, DOI: 10.1002/ece3.4816 (2019).

67. Liozon, R., Badeck, F.-W., Genty, B., Meyer, S. & Saugier, B. Leaf photosynthetic characteristics of beech (fagus sylvatica) saplings during three years of exposure to elevated co2 concentration. *Tree Physiol.* **20**, 239–247 (2000).

68. Robakowski, P., Montpied, P. & Dreyer, E. Responses to temperature and shade in abies alba seedlings from diverse provenances. *Scand. journal forest research* **20**, 459–470 (2005).

69. Wohlfahrt, G. *et al.* Inter-specific variation of the biochemical limitation to photosynthesis and related leaf traits of 30 species from mountain grassland ecosystems under different land use. *Plant, Cell & Environ.* **22**, 1281–1296 (1999).

70. Bubier, J. L. *et al.* Effects of nutrient addition on leaf chemistry, morphology, and photosynthetic capacity of three bog shrubs. *Oecologia* **167**, 355–368, DOI: 10.1007/S00442-011-1998-9 (2011).

71. Way, D. A. & Sage, R. F. Elevated growth temperatures reduce the carbon gain of black spruce [picea mariana (mill.) bsp]. *Glob. Chang. Biol.* **14**, 624–636, DOI: 10.1111/j.1365-2486.2007.01513.x (2008).

72. Wullschleger, S. D. Biochemical limitations to carbon assimilation in c3 plants—a retrospective analysis of the a/ci curves from 109 species. *J. Exp. Bot.* **44**, 907–920, DOI: 10.1093/jxb/44.5.907 (1993).

73. Tomaszewski, T. & Sievering, H. Canopy uptake of atmospheric n deposition at a conifer forest: Part ii-response of chlorophyll fluorescence and gas exchange parameters. *Tellus B: Chem. Phys. Meteorol.* **59**, 493–501, DOI: 10.1111/j.1600-0889.2007.00265.x (2007).

74. Cannone, N. *et al.* The interaction of biotic and abiotic factors at multiple spatial scales affects the variability of co 2 fluxes in polar environments. *Polar Biol.* **39**, 1581–1596, DOI: 10.1007/s00300-015-1883-9 (2016).

75. Fan, Y., Zhong, Z. & Zhang, X. Determination of photosynthetic parameters vcmax and jmax for a c3 plant (spring hulless barley) at two altitudes on the tibetan plateau. *Agric. For. Meteorol.* **151**, 1481–1487, DOI: 10.1016/j.agrformet.2011.06.004 (2011).

76. Keiner, R., Gruselle, M.-C., Michalzik, B., Popp, J. & Frosch, T. Raman spectroscopic investigation of 13 co 2 labeling and leaf dark respiration of fagus sylvatica l.(european beech). *Anal. bioanalytical chemistry* **407**, 1813–1817, DOI: 10.1007/s00216-014-8446-8 (2015).

77. Smith, E. M. & Hadley, E. B. Photosynthetic and respiratory acclimation to temperature in ledum groenlandicum populations. *Arct. Alp. Res.* **6**, 13–27, DOI: 10.1080/00040851.1974.12003756 (1974).

78. Goulden, M. L. *et al.* Physiological responses of a black spruce forest to weather. *J. Geophys. Res. Atmospheres* **102**, 28987–28996, DOI: 10.1029/97JD01111 (1997).

79. Reich, P. B. *et al.* Relationships of leaf dark respiration to leaf nitrogen, specific leaf area and leaf life-span: a test across biomes and functional groups. *Oecologia* **114**, 471–482, DOI: 10.1007/s004420050471 (1998).

80. Ueyama, M. *et al.* Leaf-and ecosystem-scale photosynthetic parameters for the overstory and understory of boreal forests in interior alaska. *J. Agric. Meteorol.* **74**, 79–86, DOI: 10.2480/agrmet.D-17-00031 (2018).

81. Stoner, E. R., Baumgardner, M., Biehl, L. & Robinson, B. *Atlas of soil reflectance properties* (Purdue University West Lafayette, 1980).

82. Hashimoto, A., Segah, H., Yulianti, N., Naruse, N. & Takahashi, Y. A new indicator of forest fire risk for indonesia based on peat soil reflectance spectra measurements. *Int. J. Remote. Sens.* **42**, 1917–1927 (2021).

83. Schenk, H. J. & Jackson, R. B. The global biogeography of roots. *Ecol. monographs* **72**, 311–328, DOI: 10.1890/0012-9615(2002)072[0311:TGBOR]2.0.CO;2 (2002).

84. Wohlfahrt, G., Bahn, M., Horak, I., Tappeiner, U. & Cernusca, A. A nitrogen sensitive model of leaf carbon dioxide and water vapour gas exchange: application to 13 key species from differently managed mountain grassland ecosystems. *Ecol. Model.* **113**, 179–199 (1998).

85. Martinez, C. *et al.* Belowground carbon allocation patterns as determined by the in-growth soil core 13c technique across different ecosystem types. *Geoderma* **263**, 140–150 (2016).

86. Hébert, F. & Thiffault, N. The biology of canadian weeds. 146. *Rhododendron groenlandicum* (Oeder) kron and judd. *Can. J. Plant Sci.* **91**, 725–738, DOI: 10.4141/cjps2010-012 (2011).

87. Lieffers, V. & Rothwell, R. Effects of depth of water table and substrate temperature on root and top growth of picea mariana and larix lancina seedlings. *Can. J. For. Res.* **16**, 1201–1206, DOI: 10.1139/x86-214 (1986).

88. Flanagan, P. & Van Cleve, K. Microbial biomass, respiration and nutrient cycling in a black spruce taiga ecosystem. *Ecol. Bull.* 261–273 (1977).
